# Supplementary material for: ‘You have got a foreign body in there’: renal transplantation, unexpected mild-to-moderate distress and patients’ support needs: a qualitative study
Source: BMJ Open. 2020 Mar 31;10(3):e035627. doi: 10.1136/bmjopen-2019-035627 (PMC7170619; doi:10.1136/bmjopen-2019-035627)
Supplement: Author's manuscript [file bmjopen-2019-035627.draft_revisions.pdf]

BMJ Open is committed to open peer review. As part of this commitment we make the peer review history of every article we publish publicly available.

When an article is published we post the peer reviewers' comments and the authors' responses online. We also post the versions of the paper that were used during peer review. These are the versions that the peer review comments apply to.

The versions of the paper that follow are the versions that were submitted during the peer review process. They are not the versions of record or the final published versions. They should not be cited or distributed as the published version of this manuscript.

BMJ Open is an open access journal and the full, final, typeset and author-corrected version of record of the manuscript is available on our site with no access controls, subscription charges or pay-per-view fees (http://bmjopen.bmj.com).

If you have any questions on BMJ Open's open peer review process please email info.bmjopen@bmj.com

BMJ Open

"You've got a foreign body in there": Renal transplantation, unexpected distress and patients' support needs: a qualitative study

Journal:	*BMJ Open*
Manuscript ID	bmjopen-2019-035627
Article Type:	Original research
Date Submitted by the Author:	08-Nov-2019
Complete List of Authors:	Jones, Janet ; University of Birmingham, Institute of Applied Health Research<br>Damery, Sarah; University of Birmingham, Institute of Applied Health Research<br>Allen, Kerry; University of Birmingham, Health Services Management Centre<br>Nicholas, Johann; Shrewsbury and Telford Hospital NHS Trust, Renal Unit<br>Baharani, Jyoti; Heart of England NHS Foundation Trust, Renal Medicine<br>Combes, Gill; University of Birmingham, Institute of Applied Health Research
Keywords:	Renal transplantation < NEPHROLOGY, Anxiety disorders < PSYCHIATRY, QUALITATIVE RESEARCH

SCHOLARONE™
Manuscripts

I, the Submitting Author has the right to grant and does grant on behalf of all authors of the Work (as defined in the below author licence), an exclusive licence and/or a non-exclusive licence for contributions from authors who are: i) UK Crown employees; ii) where BMJ has agreed a CC-BY licence shall apply, and/or iii) in accordance with the terms applicable for US Federal Government officers or employees acting as part of their official duties; on a worldwide, perpetual, irrevocable, royalty-free basis to BMJ Publishing Group Ltd ("BMJ") its licensees and where the relevant Journal is co-owned by BMJ to the co-owners of the Journal, to publish the Work in this journal and any other BMJ products and to exploit all rights, as set out in our licence.

The Submitting Author accepts and understands that any supply made under these terms is made by BMJ to the Submitting Author unless you are acting as an employee on behalf of your employer or a postgraduate student of an affiliated institution which is paying any applicable article publishing charge ("APC") for Open Access articles. Where the Submitting Author wishes to make the Work available on an Open Access basis (and intends to pay the relevant APC), the terms of reuse of such Open Access shall be governed by a Creative Commons licence – details of these licences and which Creative Commons licence will apply to this Work are set out in our licence referred to above.

Other than as permitted in any relevant BMJ Author's Self Archiving Policies, I confirm this Work has not been accepted for publication elsewhere, is not being considered for publication elsewhere and does not duplicate material already published. I confirm all authors consent to publication of this Work and authorise the granting of this licence.

For peer review only

"You've got a foreign body in there": Renal transplantation, unexpected distress and patients' support needs: a qualitative study

Short title: Unexpected distress in renal transplant patients

Janet Jones*[1], Sarah Damery[1], Kerry Allen[2], Johann Nicholas[3^], Jyoti Baharani[4], and Gill Combes[1],

[1]Institute of Applied Health Research, University of Birmingham, Birmingham UK.

[2]Health Services Management Centre, University of Birmingham, Birmingham, UK.

[3]Renal Unit, New Cross Hospital, Royal Wolverhampton NHS Trust, Wolverhampton,

[4]Renal Unit, Birmingham Heartlands Hospital, University Hospital Birmingham NHS Foundation Trust, Birmingham, UK.

*Corresponding author at: Institute of Applied Health Research, University of Birmingham, Edgbaston, Birmingham B15 2TT, UK.

E mail address: j.e.jones@bham.ac.uk, Tel number: 0121 414 7661.

^ Current affiliation: Renal Unit, Royal Shrewsbury Hospital, Shrewsbury, UK

Word count: 3317

For peer review only - http://bmjopen.bmj.com/site/about/guidelines.xhtml

ABSTRACT

Objective: To explore why transplant patients experience mild-moderate distress and what support they may need.

Design: Qualitative study using individual in-depth interviews.

Setting: Four NHS Trusts in the Midlands, UK.

Participants: Fifteen renal transplant patients meeting the criteria for mild to moderate distress from their responses to emotion thermometers.

Main outcome measures: identification of the reasons for distress and support options acceptable to renal transplant patients.

Results: Three themes were interpreted from the data: 'I'm living with a "foreign body" inside me', 'why am I distressed?' and, 'different patients want different support'. Following their transplant, participants felt that they should be happy and content, but this was often not the case. They described a range of feelings about their transplant, such as: uncertainty about the lifespan of their new kidney, fear of transplant failure or fear of the donor having health conditions that may transfer to them. A few experienced survivors' guilt when others they had met at the dialysis unit had not received a transplant or because someone had died to enable them to receive the transplant. No longer having regular contact with the renal unit made participants feel isolated. Some participants did not initially attribute the source of their distress to their transplant. Participants' preferred support for their distress and their preferences about who should deliver it varied from peer support to seeing a psychologist.

For peer review only - http://bmjopen.bmj.com/site/about/guidelines.xhtml

Conclusion: Raising the issue of post-transplant mild-to-moderate distress with patients and encouraging them to think about and plan coping strategies pre-transplant may prove beneficial for the patient and healthcare provider. Patients should be able to choose from a variety of support options.

ARTICLE SUMMARY

Strengths and limitations of this study

- This is the first study designed to explore the issue of mild-to-moderate distress in renal transplant patients.
- The Interviews were a sub-set of data from a larger study.
- To reduce bias on the basis of the services offered to liver patients multiple sites with different organisation and delivery of services were selected.
- The diversity of participant characteristics supports the representativeness of the findings to this patient group.

For peer review only

For peer review only - http://bmjopen.bmj.com/site/about/guidelines.xhtml

INTRODUCTION

At the end of 2017, 63,162 adults were receiving renal replacement therapy in the UK [1], and in the same year 3,462 transplants were performed.[1] Patients with end stage renal disease (ESRD) can experience distress and distress is associated with lower quality of life and greater treatment burden.[2] The level of emotional distress increases as the health of a patient with ESRD declines [3] and the prevalence of depression and anxiety in patients with ESRD is approximately four times higher than in the general adult population.[4] Damery et al 2019 [5] reported that more than a third of renal dialysis patients suffer emotional distress. If suffering from mild-to-moderate distress – the unpleasant feelings or emotions that may interfere with patients' ability to cope with their kidney transplant, its physical symptoms and its treatments - patients may withdraw from treatment, be non-compliant with medication and diet [6] or be reluctant to engage in pre-renal replacement therapy (RRT) education and support.[7]

Although not a cure, for patients with ESRD, receiving a transplant provides the best chance of improving their quality of life and removing the burden of undergoing long-term dialysis treatment.[8 9] Nevertheless, research has shown that although receiving a transplant is the main goal for ESRD patients who are eligible for transplantation, they may continue to experience distress. For example, fear of the transplant failing can affect patient distress levels.[8] There is also evidence that patients may experience ongoing physical symptoms such as fatigue after receiving a transplant, and that there is substantial burden associated with taking regular immunosuppressant medication to lower the likelihood of graft rejection.[10] UK health

For peer review only - http://bmjopen.bmj.com/site/about/guidelines.xhtml

policies highlight the importance of addressing the emotional and psychological needs of renal transplant patients [11] and the Department of Health and NHS England advocate treating mental health on an equal footing to that of physical health by incorporating it into care pathways.[12] Nevertheless, evidence suggests that distress, coping, and adjustment in transplant patients largely go undiagnosed or ignored and remain untreated.[13] Currently, there is little evidence on mild-to-moderate distress in transplant patients and further research is required in order to understand the psychological and emotional effects of a transplant.[9]

As an element of a larger mixed-methods study with patients and staff,[14] this paper reports the findings from a qualitative study with renal transplant patients. The aims were to explore why transplant patients experience distress and what support they may need.

METHODS

The detailed methods for the study are in the published protocol.[14] In brief, participants were recruited from four National Health Service (NHS) hospitals in the West Midlands, UK. The chosen sites provided maximum diversity in patient demographics, catchment size, urban and rural locations plus the organisation of psychological support services, table 1.

Table 1. Summary of recruitment sites

Site	Size	Catchment area	On-site renal psychologist

For peer review only

For peer review only - http://bmjopen.bmj.com/site/about/guidelines.xhtml

1	Small	Urban inner city with sizable Black, Asian and minority ethnic (BAME) population	No
2	Large	Urban inner city with sizable BAME population	Yes
3	Medium	Urban with surrounding rural districts majority white population	No
4	Large	Urban with surrounding rural districts majority white population	Yes

For peer review only

To be eligible for recruitment to the qualitative study, patients had to be categorised as having mild-to-moderate distress based on their responses to the distress thermometer [15] included in the wider study questionnaire. A researcher contacted all participants meeting the purposive sampling criteria (age, gender and ethnicity) to ask if they were willing to take part in an interview, a consent form and participant information sheet explaining the purpose of the study and what participation would involve, was sent to those expressing interest. As well as assuring patient confidentiality, the patient information sheet provided contact details for appropriate clinical staff the participants could contact if they felt distressed or upset and would like support. The following week a researcher contacted the potential participants to confirm participation and arrange an interview date and time.

Patient interviews took place between March 2016 and May 2017 and were conducted by two experienced researchers (both female and educated to masters level) employed by the University of Birmingham. Neither researcher had experience (personally or professionally) of the topic area and none of the participants knew the researchers. Interviews were either face-to-face (at a location chosen by the

For peer review only - http://bmjopen.bmj.com/site/about/guidelines.xhtml

participant, either a quiet room at the hospital or at the patients home) or over the telephone and lasted between 30 - 60 minutes. The interviews were in-depth and semi-structured allowing the exploration of key issues without being overly prescriptive about content and direction. All participants provided signed written informed consent prior to their interview. Patient advisors, renal clinicians and the current literature helped with the design of the topic guide, see box 1 for the areas covered by the topic guide. Both researchers made field notes following each interview. Interviews were audio-recorded and transcribed verbatim by a professional transcribing service. Transcripts were checked against the recordings for accuracy. Participants did not have the opportunity to review their interview transcript.

a) Experience of emotional difficulties and needs linked to their illness and/or treatment, when and for how long.
b) Language used around emotional difficulties and needs, and its meaning.
c) Whether and how emotional needs have been recognised and supported by renal staff, when and by whom.
d) What, if any, support used, when and why.
e) Likes and dislikes of support used.
f) Support would have liked/would want in future, when, and from whom.
g) Key elements would want included in an emotional support intervention

Box 1. Areas covered by the topic guide

Analysis combined aspects of grounded theory [16] and thematic analysis.[17] Interviews were initially analysed inductively using the open coding and constant comparison aspects of grounded theory. The initial coding framework was developed by JJ and CG and was appropriately refined following comparison and discussion. Transcripts were coded using NVivo 11. For data that did not fit existing themes, new codes

For peer review only - http://bmjopen.bmj.com/site/about/guidelines.xhtml

were developed or existing ones revised until all data were coded by theme. The research team discussed the role of reflexivity and our potential influence on the data and its interpretation.

RESULTS

Fifteen renal transplant patients aged between 30 and ≥70 years were recruited across the four sites, see tables 2 and 3.

Table 2. Summary of participant characteristics

		**N (%)**
**Gender**	Male	7 (47)
	Female	8 (53)
**Age**	30-39	3 (20)
	40-49	2 (13)
	50-59	4 (27)
	60-69	4 (27)
	≥70	2 (13)
**Ethnicity**	White/British	9 (60)
	Indian	4 (27)
	Caribbean	2 (13)
**Length of time on dialysis prior to transplant**	No dialysis	1 (7)
	0 – 3 years	5 (33)
	4 – 9 years	0 (0)
	≥ 10 years	3 (20)
	Unknown	6 (40)

Table 3. Individual participant characteristics

Three overarching themes were interpreted from the data. See table 4 for a summary of the themes and sub-themes:

For peer review only - http://bmjopen.bmj.com/site/about/guidelines.xhtml

Table 4. Themes and sub-themes

Theme	Sub-themes
I'm living with a "foreign body" inside me	Fear/feelings about the kidney itself
	Survivors' guilt
	Feelings of isolation
	Impacts of medication
Why am I distressed?	Initial feelings following transplant
	Association of distress to the transplant
	Coping with distress
Different patients want different types of support	Lack of information about transplants and support
	Preferred types of support and, who should deliver it

For peer review only

I'm living with a "foreign body" inside me

Fear/feelings about the kidney itself

Living with a transplanted kidney and its associated treatments can evoke many different feelings and difficulties for patients. For many there is the fear and uncertainty of how long the transplant will last: participants talked about their transplant having a finite life and how this knowledge made them worry about what would happen in the future. Three participants had experienced a failed transplant and others understood the status of their transplant could change at any time:

> *"A lot of people I knew before when I was on dialysis who had transplants, they had rejections and all sorts of things. So that's passing through your mind all the while".* (M225).

Among some patients, their fear had escalated the longer they had been with a transplant, particularly after their transplant exceeded the average lifespan. Some patients thought the longevity of a transplanted kidney was something of a lottery, which was percevied to be outside of their control and could therefore cause

For peer review only - http://bmjopen.bmj.com/site/about/guidelines.xhtml

distress. A few patients seemed emotionally affected by feeling they had a "foreign body" inside them (M225). One male patient talked about the possible consequences for his character of having received a female kidney. Another was anxious that his transplanted kidney might have come from someone with other health problems:

> *"When I got it I started to get awful thoughts at night. 'How did this person die, how old were they, did they have anything else, could they possibly have been HIV positive? Could there have been any other things that were underlying that may come forward later on?' and I still get those sort of thoughts at the moment".* (M1141).

Survivors' guilt

Although a transplant is the gold standard treatment for ESRD, some participants mentioned feeling guilty about being distressed and some worried this may make them appear ungrateful. They felt they should be happy because they were lucky to receive a transplant: "*but you, at the same time you've got no reason to feel like that".* (M389). This guilt and the fear of appearing ungrateful prevented some from seeking help when they needed it and stopped them from moving forward with their lives. Some participants worried about the donor, what happened to them and the family they left behind and others had survivors' guilt because they had received their transplant before others who had been waiting longer:

> *"When I had my transplant I felt, I suppose what you'd call it is like in a sense survivors guilt…..And because I had my transplant so quickly I just felt this huge amount of guilt because I thought all those patients that I had met at* [hospital] *and they've been on the*

For peer review only - http://bmjopen.bmj.com/site/about/guidelines.xhtml

list for such a long time, and I thought 'what's the difference between me and them?'". (F413).

Feelings of isolation

There was a perception amongst several of the participants that after receiving a transplant they were no longer part of the renal unit. They talked about feeling 'cut-off' and 'abandoned' with minimal contact. One patient explained how there had been no contact with his renal unit for several years following his transplant. Patients that had transitioned from in-centre haemodialysis (HD) seemed to feel this loss acutely - they missed the supportive relationship of staff and patients in the dialysis unit:

"Once you're transplanted, you don't really have anything to do as such with the renal unit……the only time I actually only ever was involved with the renal unit was when I was on dialysis. So that sort of like support was gone". (F726).

Some patients experienced feelings of isolation because of the lack of understanding about their disease and its treatments among family, friends and society in general. They believed only fellow transplant patients understood their feelings:

"It helped the fact talking to a stranger about it. You know, the wife couldn't understand why I was like it and that, but obviously the stranger could because she knew the experience of it all". (M369).

For peer review only - http://bmjopen.bmj.com/site/about/guidelines.xhtml

For peer review only

Impacts of medication

Feeling down and finding their situation difficult to deal with was widespread amongst the participants. These feelings were often present when participants were dealing with the physical side effects of the anti-rejection medications such as, weight gain, puffy appearance and excessive hair growth. Other peoples' reactions to the physical changes were often difficult to deal with:

> *"You know like the new face and excessive hair ….. and I found that I think the most difficult for me was that change and unfortunately when I went back to school I had a bit of , a bit of bullying went on, because of physically changing so much from what I was previously as well".* (F687).

Participants explained how when they were on dialysis they felt in control of their body, their treatment and their lives but following transplant many felt they no longer had command of their body or life in general and yearned to take back control. Such as, not wanting to leave the house because of lack of bladder control. For the following patient the feeling of helplessness ended with her sabotaging her treatment:

> *"So the reason my kidney failed was because I felt like I didn't have much control, so I kind of stopped taking my pills, my immunosuppressant. I stopped taking them for a while".* (F413).

Why am I distressed?

For peer review only - http://bmjopen.bmj.com/site/about/guidelines.xhtml

Initial feelings following transplant

Following their transplant, participants did not expect to experience distress because they regarded a transplant as the best treatment option for an improved quality of life. Some patients had waited many years for the opportunity to receive the ‘gift’ of a transplant and had been optimistic:

> *“I’m coming at it first time with an expectation that once you get a transplant and you start feeling better again, life is rosy, life can get back to normal”.* (M389).

Association of distress to the transplant

Whilst experiencing distress, many participants did not initially relate it to their transplant. The data suggest that the reasons for this were two-fold. Firstly, following a transplant there is an expectation from family, friends, clinicians and society in general that life will return to ‘normal’ and be ‘wonderful’ again. Secondly, renal staff did not forewarn the participants they might experience distress after receiving a transplant. Some only made the connection after agreeing to take part in this research study:

> *“I never did link it to – there was something in your paperwork that I had. I thought I’ve never associated it with the kidney operation”.* (M369).

Coping with distress

For peer review only - http://bmjopen.bmj.com/site/about/guidelines.xhtml

For peer review only

Renal disease and its lifecycle can make it hard for patients to be positive about their transplant. Several participants explained how it was difficult to cope with their emotions, to move on with their lives and how they perceived family and friends, in particular, were defining them by their health condition:

> *"(Sighs) Ah, again I suppose it comes back to the fact that I don't want to like, yes like I know I've got an illness, but I don't want to be that's who I am like. You know that's what I'm all about sort of thing".* (F726).

The majority of participants developed ways of accepting post-transplant life and found ways to cope with their distress. For some, distractions in the form of hobbies and pastimes such as gardening or reading helped them to adjust and for others it was important to maintain a positive outlook about their transplant:

> *"I've always had this positive outlook on the transplant and that."* (M225).

Different patients want different types of support

Lack of information about transplants and support

Many participants wished the renal team had explained to them the possibility of experiencing post-transplant distress and lamented the lack of information about this and the lack of available support. Many felt that "forewarned is forearmed" and were upset they missed the opportunity to plan, in advance,

For peer review only - http://bmjopen.bmj.com/site/about/guidelines.xhtml

coping strategies. This lack of information provision was associated with a lack of continuity of care, and participants felt that staff regarded transplant patients as a lower priority compared to those on dialysis:

> *"Time is precious, resources are scarce, I suspect that probably it never feels as though it's a big priority".* (M389).

Preferred types of support and, who should deliver it

There were, however, diverse views amongst the participants about the types of support they would like to receive, and who should deliver it. For example, one participant described how she thought talking to other patients might have provided the support she sought.

> *"So with other patients in your situation, so that you could liaise with each other to see what new life is all about".* (F494).

There were mixed views about the role of healthcare professionals (HCPs), with some suggesting that HCPs should be more proactive in identifying distress in renal transplant patients and directing them to appropriate sources of help and support. Whereas others did not see this as the role of the renal team – they are there to provide medical assistance and advice not psychological and emotional support. Many suggested that specialist psychological services should be available as in integral part of care for renal transplant patients:

> *"I think you need psychologists as part of the renal team, a psychologist with renal expertise".* (F781).

For peer review only - http://bmjopen.bmj.com/site/about/guidelines.xhtml

For peer review only

However, because of the sensitive and personal nature of distress or for fear of showing weakness there were those who were reluctant to talk about their distress to anyone, making it difficult for HCPs to assess the support needs of these patients:

> *"You know, it might have been something to do with medication – I'm just guessing. But I should have told them but as I say, I mean it's not the sort of thing I tell people"* (M369).

For peer review only

DISCUSSION

This research has highlighted the complex relationship that renal transplant patients have with their new kidney, their surprise at the source of their distress and the diverse opinions on the types of support they would like to help them through their distress. Current research has shown that the prevalence of distress in renal transplant patients is 25% and although this is lower than the 33% of dialysis patients, it is still substantial and shows that many transplant patients experience ongoing issues.[5] There is a range of reasons why despite all expectations of living an improved life, some patients became uneasy with their transplanted kidney and why some did not associate their distress with their transplant and/or immunosuppressant medication.

Patients found it difficult to accept their new kidney because of a number of different situations including: the side effects of medication, fear of transplant rejection and

For peer review only - http://bmjopen.bmj.com/site/about/guidelines.xhtml

feeling obliged to make the most of life because of the “gift” of a transplant. It has been reported that although fear of rejection may be the biggest stressor immediately after transplant, this fear subsides with time.[8] However, our findings do not reflect this: the majority of our participants, regardless of time since transplant, reported some level of emotional distress.[5] Emotional problems such as depression, anxiety, stress, and concerns about body image are known indicators of poor medication adherence.[3 6] and as discussed, may result in patients wishing they were back on dialysis and in some cases sabotaging their treatment. Gill,[8] Suzuki [18] and Fox [19] found that those receiving a kidney from a living donor had a vested interest in each other’s well-being and were motivated to comply with their medication regimes and enjoyed an improved quality of life. For those receiving a cadaver kidney, thoughts about who their kidney came from were forefront in their mind and for some this had a negative effect on their relationship with their kidney – making them feel as though they had a “foreign body” inside them. The emotional distress patients feel may be down to unrealistically high expectations of life post-transplant and there is a need for these expectations to be managed by renal services.[8 20 21]

Some of our participants did not explicitly link their distress to their transplant or the immunosuppressive medication. To our knowledge, there is no discussion of this aspect of distress and renal transplantation in the current literature. The reason for this lack of association may be that patients naively perceive that by having a transplant their quality of life will improve quickly and they will return to a normal life.[22] Given that patients receive little, if any, information about post-transplant distress from HCPs [3] this is not surprising. Consequently, patients are often ill-prepared and feel helpless when trying to cope with their distress.[23] Evidence has

For peer review only

For peer review only - http://bmjopen.bmj.com/site/about/guidelines.xhtml

shown that improving coping skills,[24] education before and after transplantation,[25] and active information seeking by patients can have a beneficial effect on patients' medical and psychological problems.[8 20 26] The National Institute for Health and Clinical Excellence (NICE) advocate keeping patients informed at all stages of treatment and encourage the promotion of self-care and self-management skills.[27] However, HCPs can find it difficult to recognise distress or anxiety in patients and are unsure at which point in the disease trajectory to discuss this with their patients.[28] When a patient is informed, it empowers them to take control of their condition and having control can itself lower the chances of distress.[6] It is therefore important that HCPs provide and share information and discuss all possible outcomes and coping strategies at the appropriate time in the treatment pathway.[3 21 23]

Not all renal transplant patients will experience distress and not all of those who do will want to receive support. This research has shown that some patients do not wish to talk about their feelings for fear of appearing ungrateful or weak, making it difficult for HCPs to understand, assess and procure appropriate support.[28] Even when patients do want to talk about their feelings, there is "no one size fits all" solution to the provision of support.[29] Different patients have different emotional needs indicating that any support offered to patients should be individualised in order to meet this variance of need.[30]

A limitation of this study is that the interview data analysed for this research is a sub-set of a larger set of data. Although the 15 in-depth interviews provided sufficient

For peer review only - http://bmjopen.bmj.com/site/about/guidelines.xhtml

data to answer the research questions, it is important for future research to explore these areas with a more diverse and carefully stratified sample.

CONCLUSIONS

Our research has highlighted a number of points: firstly, it is important to talk to patients and their families pre-transplant about the possibility of experiencing mild-to-moderate post-transplant distress. Secondly, patients should be encouraged to think about potential coping strategies and finally, transplant patients with mild-to-moderate distress should be able to choose from a variety of support options: peer support; HCPs with augmented skills in detecting and managing distress; access to psychology services.

Acknowledgements: Francesca Taylor and Elaine Kidney conducted the interviews. Pamela Nayyar provided administrative support for this research. The patient and public involvement group of the CLAHRC West Midlands long-term conditions theme and a renal patient advisory group provided advice on the design of the study and the data collection tools. Finally, a huge thank you goes out to all of the participants who gave up their time to talk to us.

Author Contributors: JJ: Conceptualisation (for this sub-study), formal analysis, interpretation of data, methodology, validation, writing (original draft); GC: formal analysis, validation, writing (critical review and editing); SD, & KA: interpretation of

For peer review only - http://bmjopen.bmj.com/site/about/guidelines.xhtml

data, writing (critical review and editing), JN & JB: Conceptualisation, validation, writing (critical review and editing). All authors have approved the final version.

Funding: This research was funded by the National Institute for Health Research Collaboration for Leadership in Applied Health Research and Care West Midlands (NIHR CLAHRC WM), now recommissioned as NIHR Applied Research Collaboration West Midlands. The views expressed in this publication are those of the author(s) and not necessarily those of the NIHR or the Department of Health and Social Care.

Competing interests: -None

Ethical approval: The Coventry and Warwickshire Research Ethics Committee granted approval for this and the wider study in October 2015 [Ref 15/WM/0288]. The Research Governance office at each of the participating hospital trusts also gave approval for the study.

Data availability: The research data is confidential. Participants did not give consent to share their data and the ethical requirements of the study do not allow us to share the study data.

REFERENCES

For peer review only

1. UK Renal Registry 21st Annual Report - data to 31/12/2017. Bristol, UK, 2019.
2. Soni RK. Health-related quality of life outcomes in chronic kidney disease. *Curr Opin Nephrol Hypertens*;19(2):153-60. doi: https://doi.org/10.1097/MNH.0b013e328335f939
3. Schell JO, Patel UD, Steinhauser KE, et al. Discussions of the kidney disease trajectory by elderly patients and nephrologists: a qualitative study. *American journal of kidney diseases : the official journal of the National Kidney Foundation* 2012;59(4):495-503. doi: https://doi.org/10.1053/j.ajkd.2011.11.023 [published Online First: 01/04]
4. Murtagh FEM, Addington-Hall J, Higginson IJ. The Prevalence of Symptoms in End-Stage Renal Disease: A Systematic Review. *Advances in Chronic Kidney Disease* 2007;14(1):82-99. doi: https://doi.org/10.1053/j.ackd.2006.10.001
5. Damery S, Brown C, Sein K, et al. The prevalence of mild-to-moderate distress in patients with end-stage renal disease: results from a patient survey using the emotion thermometers in four hospital Trusts in the West Midlands, UK. *BMJ open* 2019;9(5):e027982. doi: https://doi.org/10.1136/bmjopen-2018-027982
6. Cukor D, Rosenthal DS, Jindal RM, et al. Depression is an important contributor to low medication adherence in hemodialyzed patients and transplant recipients. *Kidney Int* 2009;75(11):1223-29. doi: https://doi.org/10.1038/ki.2009.51
7. Combes G, Allen K, Sein K. Evaluation of case study sites for home therapies for people with chronic kidney disease Birmingham: University of Birmingham: West Midlands Central Health Innovation and Education Cluster; 2013 [Available from: http://www.birmingham.ac.uk/research/activity/mds/projects/WMC-HIEC/publications.aspx accessed May 19.
8. Gill P. Stressors and coping mechanisms in live-related renal transplantation. *J Clin Nurs* 2012;21(11-12):1622-31. doi: https://doi.org/10.1111/j.1365-2702.2012.04085.x
9. Kohlsdorf M. Coping Strategies Adopted by Patients With Chronic Kidney Disease in Preparation for Transplant. *Psychol Comm health* 2015;4(1):27-38. doi: https://doi.org/10.5964/pch.v4i1.103 [published Online First: 2015-03-31]
10. Cukor D, Newville H, Jindal R. Depression and immunosuppressive medication adherence in kidney transplant patients. *General hospital psychiatry* 2008;30(4):386-7. doi: https://doi.org/10.1016/j.genhosppsych.2007.12.003 [published Online First: 2008/07/01]
11. Department of Health, The National Service Framework for Renal Services Part 2: Chronic kidney disease, acute renal failure and end of life care. London: Department of Health 2005.
12. Department of Health, Closing the gap: priorities for essential change in mental health. London: Department of Health 2014
13. Spencer BW, Chilcot J, Farrington K. Still sad after successful renal transplantation: are we failing to recognise depression? An audit of depression screening in renal graft recipients. *Nephron Clinical practice* 2011;117(2):c106-12. doi: https://doi.org/10.1159/000319657 [published Online First: 2010/08/10]
14. Taylor F, Taylor C, Baharani J, et al. Integrating emotional and psychological support into the end-stage renal disease pathway: a protocol for mixed methods research to identify patients' lower-level support needs and how these can most effectively be addressed. *BMC nephrology* 2016;17(1):111. doi: https://doi.org/10.1186/s12882-016-0327-2 [published Online First: 2016/08/04]
15. Mitchell AJ, Baker-Glenn EA, Granger L, et al. Can the Distress Thermometer be improved by additional mood domains? Part I. Initial validation of the Emotion Thermometers tool. *Psycho-Oncology* 2010;19(2):125-33. doi: https://doi.org/10.1002/pon.1523
16. K C, L B. Qualitative interviewing and grounded theory analysis. In: Gubrium JF, Holstein AB, McKinney KD, eds. The SAGE Handbook of interview research: The Complexity of the Craft. California, USA: Sage publications 2012.
17. Braun V, Clarke V. Successful Qualitative Research: a practical guide for beginners. Thousand Oaks, CA: Sage 2013:174-179.

For peer review only - http://bmjopen.bmj.com/site/about/guidelines.xhtml

18. Suzuki A, Kenmochi T, Maruyama M, et al. Changes in quality of life in deceased versus living-donor kidney transplantations. *Transplant Proc* 2012;44(1):287-9. doi: https://doi.org/10.1016/j.transproceed.2011.11.056 [published Online First: 2012/02/09]
19. Fox RC. Through the lenses of biology and sociology: Organ replacement. In: Williams SJ, Birke L, Bendelow GA, eds. Debating Biology: Sociological reflection on health, medicine and society. London: Routledge 2003.
20. Chilcot J, Spencer BWJ, Maple H, et al. Depression and Kidney Transplantation. *Transplantation* 2014;97(7):717-21. doi: https://doi.org/10.1097/01.TP.0000438212.72960.ae
21. Pinter J, Hanson CS, Craig JC, et al. 'I feel stronger and younger all the time'—perspectives of elderly kidney transplant recipients: thematic synthesis of qualitative research. *Nephrol Dial Transplant* 2016;31(9):1531-40. doi: https://doi.org/10.1093/ndt/gfv463
22. Boaz A, Morgan M. Working to establish 'normality' post-transplant: a qualitative study of kidney transplant patients. *Chronic illness* 2014;10(4):247-58. doi: https://doi.org/10.1177/1742395313504789 [published Online First: 2013/09/28]
23. Cormier NR, Gallo-Cruz SR, Beard RL. Navigating the new, transplanted self: how recipients manage the cognitive risks of organ transplantation. *Sociology of health & illness* 2017;39(8):1496-513. doi: https://doi.org/10.1111/1467-9566.12610 [published Online First: 2017/10/11]
24. Been-Dahmen JMJ, Beck DK, Peeters MAC, et al. Evaluating the feasibility of a nurse-led self-management support intervention for kidney transplant recipients: a pilot study. *BMC nephrology* 2019;20(1):143. doi: https://doi.org/10.1186/s12882-019-1300-7 [published Online First: 2019/04/29]
25. Curcani M, Tan M. the factors affecting the qualilty of life of patients who have undergone kidney transplants *Pakistan Journal of Medicine* 2012;27(5):1092-97.
26. Muehrer RJ, Becker BN. Life after transplantation: new transitions in quality of life and psychological distress. *Semin Dial* 2005;18(2):124-31. doi: https://doi.org/10.1111/j.1525-139X.2005.18214.x [published Online First: 2005/03/18]
27. National Institute for Health and Clinical Excellence, CG138 Patient experience in NHS services: improving the experience of care for people using adult NHS services Manchester: National Institute for Health and Clinical Excellence 2012 [Available from: http://guidance.nice.org.uk/cg138 accessed July 2019.
28. Damery S, Sein K, Combes G. The prevalence, experience and management of mild to moderate distress in patients with end stage renal disease: results from a multi-centre, mixed methods research study: NIHR CLAHRC-WM, University of Birmingham, 2019.
29. Been-Dahmen JMJ, Grijpma JW, Ista E, et al. Self-management challenges and support needs among kidney transplant recipients: A qualitative study. *Journal of advanced nursing* 2018;74(10):2393-405. doi: https://doi.org/10.1111/jan.13730 [published Online First: 2018/06/06]
30. Grijpma JW, Tielen M, van Staa AL, et al. Kidney transplant patients' attitudes towards self-management support: A Q-methodological study. *Patient Educ Couns* 2016;99(5):836-43. doi: https://doi.org/10.1016/j.pec.2015.11.018 [published Online First: 2015/12/20]

For peer review only - http://bmjopen.bmj.com/site/about/guidelines.xhtml

COREQ (COnsolidated criteria for REporting Qualitative research) Checklist

A checklist of items that should be included in reports of qualitative research. You must report the page number in your manuscript where you consider each of the items listed in this checklist. If you have not included this information, either revise your manuscript accordingly before submitting or note N/A.

Topic	Item No.	Guide Questions/Description	Reported on Page No.
**Domain 1: Research team and reflexivity**			
Personal characteristics			
Interviewer/facilitator	1	Which author/s conducted the interview or focus group?	6
Credentials	2	What were the researcher's credentials? E.g. PhD, MD	6
Occupation	3	What was their occupation at the time of the study?	6
Gender	4	Was the researcher male or female?	6
Experience and training	5	What experience or training did the researcher have?	6
Relationship with participants			
Relationship established	6	Was a relationship established prior to study commencement?	6
Participant knowledge of the interviewer	7	What did the participants know about the researcher? e.g. personal goals, reasons for doing the research	6
Interviewer characteristics	8	What characteristics were reported about the inter viewer/facilitator? e.g. Bias, assumptions, reasons and interests in the research topic	6
**Domain 2: Study design**			
Theoretical framework			
Methodological orientation and Theory	9	What methodological orientation was stated to underpin the study? e.g. grounded theory, discourse analysis, ethnography, phenomenology, content analysis	Referred to prc
Participant selection			
Sampling	10	How were participants selected? e.g. purposive, convenience, consecutive, snowball	6
Method of approach	11	How were participants approached? e.g. face-to-face, telephone, mail, email	6
Sample size	12	How many participants were in the study?	8
Non-participation	13	How many people refused to participate or dropped out? Reasons?	Data not avalal
Setting			
Setting of data collection	14	Where was the data collected? e.g. home, clinic, workplace	7
Presence of non-participants	15	Was anyone else present besides the participants and researchers?	Data not availa
Description of sample	16	What are the important characteristics of the sample? e.g. demographic data, date	8
Data collection			
Interview guide	17	Were questions, prompts, guides provided by the authors? Was it pilot tested?	7
Repeat interviews	18	Were repeat inter views carried out? If yes, how many?	N/A
Audio/visual recording	19	Did the research use audio or visual recording to collect the data?	7
Field notes	20	Were field notes made during and/or after the inter view or focus group?	7
Duration	21	What was the duration of the inter views or focus group?	7
Data saturation	22	Was data saturation discussed?	19
Transcripts returned	23	Were transcripts returned to participants for comment and/or	7

For peer review only

Topic	Item No.	Guide Questions/Description	Reported on Page No.
		correction?	
**Domain 3: analysis and findings**			
Data analysis			
Number of data coders	24	How many data coders coded the data?	7
Description of the coding tree	25	Did authors provide a description of the coding tree?	9
Derivation of themes	26	Were themes identified in advance or derived from the data?	7
Software	27	What software, if applicable, was used to manage the data?	7
Participant checking	28	Did participants provide feedback on the findings?	7
Reporting			
Quotations presented	29	Were participant quotations presented to illustrate the themes/findings? Was each quotation identified? e.g. participant number	9-16
Data and findings consistent	30	Was there consistency between the data presented and the findings?	9-16
Clarity of major themes	31	Were major themes clearly presented in the findings?	9-16
Clarity of minor themes	32	Is there a description of diverse cases or discussion of minor themes?	10

Developed from: Tong A, Sainsbury P, Craig J. Consolidated criteria for reporting qualitative research (COREQ): a 32-item checklist for interviews and focus groups. *International Journal for Quality in Health Care*. 2007. Volume 19, Number 6: pp. 349 – 357

Once you have completed this checklist, please save a copy and upload it as part of your submission. DO NOT include this checklist as part of the main manuscript document. It must be uploaded as a separate file.

For peer review only

BMJ Open

“You’ve got a foreign body in there”: Renal transplantation, unexpected mild-to-moderate distress and patients’ support needs: a qualitative study

Journal:	*BMJ Open*
Manuscript ID	bmjopen-2019-035627.R1
Article Type:	Original research
Date Submitted by the Author:	13-Dec-2019
Complete List of Authors:	Jones, Janet ; University of Birmingham, Institute of Applied Health Research<br>Damery, Sarah; University of Birmingham, Institute of Applied Health Research<br>Allen, Kerry; University of Birmingham, Health Services Management Centre<br>Nicholas, Johann; Shrewsbury and Telford Hospital NHS Trust, Renal Unit<br>Baharani, Jyoti; Heart of England NHS Foundation Trust, Renal Medicine<br>Combes, Gill; University of Birmingham, Institute of Applied Health Research
**Primary Subject Heading**:	Renal medicine
Secondary Subject Heading:	Qualitative research
Keywords:	Renal transplantation < NEPHROLOGY, Anxiety disorders < PSYCHIATRY, QUALITATIVE RESEARCH

SCHOLARONE™
Manuscripts

I, the Submitting Author has the right to grant and does grant on behalf of all authors of the Work (as defined in the below author licence), an exclusive licence and/or a non-exclusive licence for contributions from authors who are: i) UK Crown employees; ii) where BMJ has agreed a CC-BY licence shall apply, and/or iii) in accordance with the terms applicable for US Federal Government officers or employees acting as part of their official duties; on a worldwide, perpetual, irrevocable, royalty-free basis to BMJ Publishing Group Ltd ("BMJ") its licensees and where the relevant Journal is co-owned by BMJ to the co-owners of the Journal, to publish the Work in this journal and any other BMJ products and to exploit all rights, as set out in our licence.

The Submitting Author accepts and understands that any supply made under these terms is made by BMJ to the Submitting Author unless you are acting as an employee on behalf of your employer or a postgraduate student of an affiliated institution which is paying any applicable article publishing charge ("APC") for Open Access articles. Where the Submitting Author wishes to make the Work available on an Open Access basis (and intends to pay the relevant APC), the terms of reuse of such Open Access shall be governed by a Creative Commons licence – details of these licences and which Creative Commons licence will apply to this Work are set out in our licence referred to above.

Other than as permitted in any relevant BMJ Author's Self Archiving Policies, I confirm this Work has not been accepted for publication elsewhere, is not being considered for publication elsewhere and does not duplicate material already published. I confirm all authors consent to publication of this Work and authorise the granting of this licence.

For peer review only

"You've got a foreign body in there": Renal transplantation, unexpected mild-to-moderate distress and patients' support needs: a qualitative study

Short title: Unexpected distress in renal transplant patients

Janet Jones*[1], Sarah Damery[1], Kerry Allen[2], Johann Nicholas[3^], Jyoti Baharani[4], and Gill Combes[1],

[1]Institute of Applied Health Research, University of Birmingham, Birmingham UK.

[2]Health Services Management Centre, University of Birmingham, Birmingham, UK.

[3]Renal Unit, New Cross Hospital, Royal Wolverhampton NHS Trust, Wolverhampton,

[4]Renal Unit, Birmingham Heartlands Hospital, University Hospital Birmingham NHS Foundation Trust, Birmingham, UK.

*Corresponding author at: Institute of Applied Health Research, University of Birmingham, Edgbaston, Birmingham B15 2TT, UK.

E mail address: j.e.jones@bham.ac.uk, Tel number: 0121 414 7661.

^ Current affiliation: Renal Unit, Royal Shrewsbury Hospital, Shrewsbury, UK

Word count: 3510

For peer review only

For peer review only - http://bmjopen.bmj.com/site/about/guidelines.xhtml

ABSTRACT

Objective: To explore why transplant patients experience unexpected mild-moderate distress and what support they may need.

Design: Qualitative study using individual in-depth interviews.

Setting: Four NHS Trusts in the Midlands, UK.

Participants: Fifteen renal transplant patients meeting the criteria for mild to moderate distress from their responses to emotion thermometers.

Main outcome measures: identification of the reasons for distress and support options acceptable to renal transplant patients.

Results: Three themes were interpreted from the data: ‘I'm living with a “foreign body” inside me’, ‘why am I distressed?’ and, ‘different patients want different support’. Following their transplant, participants felt that they should be happy and content, but this was often not the case. They described a range of feelings about their transplant, such as: uncertainty about the lifespan of their new kidney, fear of transplant failure or fear of the donor having health conditions that may transfer to them. A few experienced survivors' guilt when others they had met at the dialysis unit had not received a transplant or because someone had died to enable them to receive the transplant. No longer having regular contact with the renal unit made participants feel isolated. Some participants did not initially attribute the source of their distress to their transplant. Participants' preferred support for their distress and their preferences about who should deliver it varied from peer support to seeing a psychologist.

For peer review only - http://bmjopen.bmj.com/site/about/guidelines.xhtml

Conclusion: Raising the issue of post-transplant mild-to-moderate distress with patients and encouraging them to think about and plan coping strategies pre-transplant may prove beneficial for the patient and healthcare provider. Patients should be able to choose from a variety of support options.

ARTICLE SUMMARY

Strengths and limitations of this study

- This is the first study designed to explore the issue of unexpected mild-to-moderate distress in renal transplant patients.
- The Interviews were a sub-set of data from a larger study.
- To reduce bias on the basis of the services offered to renal patients multiple sites with different organisation and delivery of services were selected.
- Participants were diverse thus the findings are representative of the wider group of renal transplant patients.

For peer review only

For peer review only - http://bmjopen.bmj.com/site/about/guidelines.xhtml

INTRODUCTION

At the end of 2017, 63,162 adults were receiving renal replacement therapy in the UK [1], and in the same year 3,462 transplants were performed.[1] Patients with end stage renal disease (ESRD) can experience distress and distress is associated with lower quality of life and greater treatment burden.[2] The level of emotional distress increases as the health of a patient with ESRD declines [3] and the prevalence of depression and anxiety in patients with ESRD is approximately four times higher than in the general adult population.[4] Damery et al 2019 [5] reported that more than a third of renal dialysis patients suffer emotional distress. If suffering from mild-to-moderate distress – the unpleasant feelings or emotions that may interfere with patients' ability to cope with their kidney transplant, its physical symptoms and its treatments - patients may withdraw from treatment, be non-compliant with medication and diet [6] or be reluctant to engage in pre-renal replacement therapy (RRT) education and support.[7]

Although not a cure, for patients with ESRD, receiving a transplant provides the best chance of improving their quality of life and removing the burden of undergoing long-term dialysis treatment.[8 9] Nevertheless, research has shown that although receiving a transplant is the main goal for ESRD patients who are eligible for transplantation, they may continue to experience distress. For example, fear of the transplant failing can affect patient distress levels.[8] There is also evidence that patients may experience ongoing physical symptoms such as fatigue after receiving a transplant, and that there is substantial burden associated with taking regular immunosuppressant medication to lower the likelihood of graft rejection.[10] UK health

For peer review only - http://bmjopen.bmj.com/site/about/guidelines.xhtml

policies highlight the importance of addressing the emotional and psychological needs of renal transplant patients [11] and the Department of Health and NHS England advocate treating mental health on an equal footing to that of physical health by incorporating it into care pathways.[12] Nevertheless, evidence suggests that distress, coping, and adjustment in transplant patients largely go undiagnosed or ignored and remain untreated.[13] Currently, there is little evidence on mild-to-moderate distress in transplant patients and further research is required in order to understand the psychological and emotional effects of a transplant.[9 14 15]

As an element of a larger mixed-methods study with patients and staff,[16] this paper reports the findings from a qualitative study with renal transplant patients. The aims were to explore why transplant patients experience distress and what support they may need.

METHODS

The detailed methods for the study are in the published protocol.[16] In brief, participants were recruited from four National Health Service (NHS) hospitals in the West Midlands, UK. The chosen sites provided maximum diversity in patient demographics, catchment size, urban and rural locations plus the organisation of psychological support services and were categorised according to the size of the catchment area. Table 1.

For peer review only - http://bmjopen.bmj.com/site/about/guidelines.xhtml

Table 1. Summary of recruitment sites

Site	Size	Catchment area	On-site renal psychologist
1	Small	Urban inner city with sizable Black, Asian and minority ethnic (BAME) population	No
2	Large	Urban inner city with sizable BAME population	Yes
3	Medium	Urban with surrounding rural districts majority white population	No
4	Large	Urban with surrounding rural districts majority white population	Yes

To be eligible for recruitment to the qualitative study, patients had to be categorised as having mild-to-moderate distress based on their responses to the distress thermometer [17] included in the wider study questionnaire. Participants indicated on the questionnaire whether or not they would be willing to take part in an interview and provided their preferred contact details. A consent form and participant information sheet explaining the purpose of the interview study and what participation would involve were sent to those expressing interest. The following week those meeting the purposive sampling criteria (age, gender and ethnicity) were contacted by a researcher (FT) to confirm participation and arrange a date and time for the interview. If participants had changed their mind they were able to withdraw from this part of the wider study. As well as assuring patient confidentiality, the patient information sheet provided contact details of appropriate clinical staff the participants could contact if they felt distressed or upset and would like support. Contacting the participants to answer their questions, to arrange the interview and to

For peer review only - http://bmjopen.bmj.com/site/about/guidelines.xhtml

remind them a few days beforehand helped to build up a rapport with each participant prior to their interview.

Patient interviews took place between March 2016 and May 2017 and were conducted by two experienced qualitative researchers (FT, EK) (both identifying as female and educated to masters level) employed by the University of Birmingham. Neither researcher had experience (personally or professionally) of the topic area and none of the participants knew the researchers. Interviews were either face-to-face (at a location chosen by the participant, either a quiet room at the hospital or at the patients home) or over the telephone and lasted between 30 - 60 minutes. The interviews were in-depth and semi-structured allowing the exploration of key issues without being overly prescriptive about content and direction. All participants provided signed written informed consent prior to their interview. Patient advisors, renal clinicians and the current literature helped with the design of the topic guide, see box 1 for the areas covered by the topic guide. Prior to the start of the interview participants were advised to let the researcher know if they needed to take a break during the interview or if they no longer wished to carry on. At the end of each interview participants were asked if they had any further comments on the topics covered or whether there were any important areas they felt had not been discussed. Both researchers made field notes following each interview. Interviews were audio-recorded and transcribed verbatim by a professional transcribing service. Transcripts were checked against the recordings for accuracy. Participants did not have the opportunity to review their interview transcript.

For peer review only

For peer review only - http://bmjopen.bmj.com/site/about/guidelines.xhtml

a) Experience of emotional difficulties and needs linked to their illness and/or treatment, when and for how long.
b) Language used around emotional difficulties and needs, and its meaning.
c) Whether and how emotional needs have been recognised and supported by renal staff, when and by whom.
d) What, if any, support used, when and why.
e) Likes and dislikes of support used.
f) Support would have liked/would want in future, when, and from whom.
g) Key elements would want included in an emotional support intervention

Box 1. Areas covered by the topic guide

Analysis combined aspects of grounded theory [18] and thematic analysis.[19] Interviews were initially analysed inductively using the open coding and constant comparison aspects of grounded theory. The initial coding framework was developed by JJ and CG and was appropriately refined following comparison and discussion. Transcripts were coded using NVivo 11. For data that did not fit existing themes, new codes were developed or existing ones revised until all data were coded by theme. The research team discussed the role of reflexivity and our potential influence on the data and its interpretation. [20 21]

Patient and public involvement

The patient and public involvement group of the NIHR CLAHRC West Midlands long-term conditions theme and a renal patient advisory group provided advice on the design of the study, the data collection tools and the selection of outcomes. All participants received a summary of the study findings.

For peer review only - http://bmjopen.bmj.com/site/about/guidelines.xhtml

RESULTS

Fifteen renal transplant patients aged between 30 and ≥70 years were recruited across the four sites, see tables 2 and 3.

Table 2. Summary of participant characteristics

		**N (%)**
**Gender**	Male	7 (47)
	Female	8 (53)
**Age**	30-39	3 (20)
	40-49	2 (13)
	50-59	4 (27)
	60-69	4 (27)
	≥70	2 (13)
**Ethnicity**	White	9 (60)
	Indian	4 (27)
	Caribbean	2 (13)
**Length of time on dialysis prior to transplant**	No dialysis	1 (7)
	0 – 3 years	5 (33)
	4 – 9 years	0 (0)
	≥ 10 years	3 (20)
	Unknown	6 (40)

Table 3. Individual participant characteristics

**ID**	**Age**	**Time on dialysis prior to transplant**
P129	40-49	No dialysis
P267	60-69	Not stated
P384	50-59	16 ½ years
P413	30-39	Not stated
P494	50-59	11 years
P687	30-39	Not stated
P726	50-59	10 years
P781	≥70	11 months
P197	40-49	1 year

For peer review only

P225	≤70	3 years
P369	60-69	Not stated
P389	50-59	3 years
P401	30-39	Not stated
P1028	60-69	6 months
P1141	60-69	Not stated

Three overarching themes were interpreted from the data. See table 4 for a summary of the themes and sub-themes:

Table 4. Themes and sub-themes

**Theme**	**Sub-themes**
I'm living with a "foreign body" inside me	Fear/feelings about the kidney itself
	Survivors' guilt
	Feelings of isolation
	Impacts of medication
Why am I distressed?	Expectations of living with a transplant
	Coping with distress
	Lack of information about transplants and support
Different patients want different types of support	

I'm living with a "foreign body" inside me

Fear/feelings about the kidney itself

Living with a transplanted kidney and its associated treatments can evoke many different feelings and difficulties for patients. For many there is the fear and uncertainty of how long the transplant will last: participants talked about their transplant having a finite life and how this knowledge made them worry about what would happen in the future. Three participants had experienced a failed transplant and others understood the status of their transplant could change at any time:

For peer review only - http://bmjopen.bmj.com/site/about/guidelines.xhtml

> *"A lot of people I knew before when I was on dialysis who had transplants, they had rejections and all sorts of things. So that's passing through your mind all the while".* (P225).

Among some patients, their fear had escalated the longer they had been with a transplant, particularly after their transplant exceeded the average lifespan. Some patients thought the longevity of a transplanted kidney was something of a lottery, which was percevied to be outside of their control and could therefore cause distress. A few patients seemed emotionally affected by feeling they had a "foreign body" inside them (M225). One male patient talked about the possible consequences for his character of having received a female kidney. Another was anxious that his transplanted kidney might have come from someone with other health problems:

> *"When I got it I started to get awful thoughts at night. 'How did this person die, how old were they, did they have anything else, could they possibly have been HIV positive? Could there have been any other things that were underlying that may come forward later on?' and I still get those sort of thoughts at the moment".* (P1141).

Survivors' guilt

Although a transplant is the gold standard treatment for ESRD, some participants mentioned feeling guilty about being distressed and some worried this may make them appear ungrateful. They felt they should be happy because they were lucky to receive a transplant: "*but you, at the same time you've got no reason to feel like that".* (M389). This guilt and the fear of appearing ungrateful prevented some from

For peer review only - http://bmjopen.bmj.com/site/about/guidelines.xhtml

seeking help when they needed it and stopped them from moving forward with their lives. Some participants worried about the donor, what happened to them and the family they left behind and others had survivors' guilt because they had received their transplant before others who had been waiting longer:

> *"When I had my transplant I felt, I suppose what you'd call it is like in a sense survivors guilt…..And because I had my transplant so quickly I just felt this huge amount of guilt because I thought all those patients that I had met at* [hospital] *and they've been on the list for such a long time, and I thought 'what's the difference between me and them?'".* (P413).

Feelings of isolation

There was a perception amongst several of the participants that after receiving a transplant they were no longer part of the renal unit. They talked about feeling 'cut-off' and 'abandoned' with minimal contact. One patient explained how there had been no contact with his renal unit for several years following his transplant. Patients that had transitioned from in-centre haemodialysis (HD) seemed to feel this loss acutely - they missed the supportive relationship of staff and patients in the dialysis unit:

> *"Once you're transplanted, you don't really have anything to do as such with the renal unit……the only time I actually only ever was involved with the renal unit was when I was on dialysis. So that sort of like support was gone".* (P726).

For peer review only - http://bmjopen.bmj.com/site/about/guidelines.xhtml

Some patients experienced feelings of isolation because of the lack of understanding about their disease and its treatments among family, friends and society in general. They believed only fellow transplant patients understood their feelings:

> *"It helped the fact talking to a stranger about it. You know, the wife couldn't understand why I was like it and that, but obviously the stranger could because she knew the experience of it all".* (P369).

Impacts of medication

Feeling down and finding their situation difficult to deal with was widespread amongst the participants. These feelings were often present when participants were dealing with the physical side effects of the anti-rejection medications such as, weight gain, puffy appearance and excessive hair growth. Other peoples' reactions to the physical changes were often difficult to deal with:

> *"You know like the new face and excessive hair ….. and I found that I think the most difficult for me was that change and unfortunately when I went back to school I had a bit of , a bit of bullying went on, because of physically changing so much from what I was previously as well".* (P687).

Participants explained how when they were on dialysis they felt in control of their body, their treatment and their lives but following transplant many felt they no longer had command of their body or life in general and yearned to take back control. Such as, not wanting to leave the house because of lack of bladder control. For the

For peer review only - http://bmjopen.bmj.com/site/about/guidelines.xhtml

following patient the feeling of helplessness ended with her sabotaging her treatment:

> *"So the reason my kidney failed was because I felt like I didn't have much control, so I kind of stopped taking my pills, my immunosuppressant. I stopped taking them for a while".* (P413).

Why am I distressed?

Expectations of living with a transplant

Following their transplant, participants did not expect to experience distress because they regarded a transplant as the best treatment option for an improved quality of life. Some patients had waited many years for the opportunity to receive the 'gift' of a transplant and had been optimistic:

> *"I'm coming at it first time with an expectation that once you get a transplant and you start feeling better again, life is rosy, life can get back to normal".* (P389).

Whilst experiencing distress, many participants did not initially relate it to their transplant. The data suggest that the reasons for this were two-fold. Firstly, following a transplant there is an expectation from family, friends, clinicians and society in general that life will return to 'normal' and be 'wonderful' again.

> *"I never did link it to – there was something in your paperwork that I had. I thought I've never associated it with the kidney operation".* (P369).

For peer review only - http://bmjopen.bmj.com/site/about/guidelines.xhtml
For peer review only

Secondly, renal staff did not forewarn the participants they might experience distress after receiving a transplant. Some only made the connection after agreeing to take part in this research study:

> *But being in the hospital and then having all sorts of side effects which is effecting me emotionally now. And that things could have been told to me before, you know, I would have prepped myself up. Some of those things could have been addressed before. It could have been helpful to have been told beforehand. (P129).*

Coping with distress

Renal disease and its lifecycle can make it hard for patients to be positive about their transplant. Several participants explained how it was difficult to cope with their emotions, to move on with their lives and how they perceived family and friends, in particular, were defining them by their health condition:

> *"(Sighs) Ah, again I suppose it comes back to the fact that I don't want to like, yes like I know I've got an illness, but I don't want to be that's who I am like. You know that's what I'm all about sort of thing".* (P726).

The majority of participants developed ways of accepting post-transplant life and found ways to cope with their distress. For some, distractions in the form

For peer review only - http://bmjopen.bmj.com/site/about/guidelines.xhtml

of hobbies and pastimes such as gardening or reading helped them to adjust and for others it was important to maintain a positive outlook about their transplant:

"I've always had this positive outlook on the transplant and that." (P225).

Lack of information about transplants and support

Many participants wished the renal team had explained to them the possibility of experiencing post-transplant distress and lamented the lack of information about this and the lack of available support. Many felt that "forewarned is forearmed" and were upset they missed the opportunity to plan, in advance, coping strategies. This lack of information provision was associated with a lack of continuity of care, and participants felt that staff regarded transplant patients as a lower priority compared to those on dialysis:

"Time is precious, resources are scarce, I suspect that probably it never feels as though it's a big priority". (P389).

Different patients want different types of support

There were, however, diverse views amongst the participants about the types of support they would like to receive, and who should deliver it. For example, one participant described how she thought talking to other patients might have provided the support she sought.

For peer review only

For peer review only - http://bmjopen.bmj.com/site/about/guidelines.xhtml

> *"So with other patients in your situation, so that you could liaise with each other to see what new life is all about".* (P494).

There were mixed views about the role of healthcare professionals (HCPs), with some suggesting that HCPs should be more proactive in identifying distress in renal transplant patients and directing them to appropriate sources of help and support. Whereas others did not see this as the role of the renal team – they are there to provide medical assistance and advice not psychological and emotional support. Many suggested that specialist psychological services should be available as in integral part of care for renal transplant patients:

> *"I think you need psychologists as part of the renal team, a psychologist with renal expertise".* (P781).

However, because of the sensitive and personal nature of distress or for fear of showing weakness there were those who were reluctant to talk about their distress to anyone, making it difficult for HCPs to assess the support needs of these patients:

> *"You know, it might have been something to do with medication – I'm just guessing. But I should have told them but as I say, I mean it's not the sort of thing I tell people"* (P369).

DISCUSSION

For peer review only - http://bmjopen.bmj.com/site/about/guidelines.xhtml
For peer review only

This research has highlighted the complex relationship that renal transplant patients have with their new kidney, their largely unanticipated experience of their distress and the diverse opinions on the types of support they would like to help them through their distress. Current research has shown that the prevalence of distress in renal transplant patients is 25% and although this is lower than the 33% of dialysis patients, it is still substantial and shows that many transplant patients experience ongoing issues.[5] There is a range of reasons why despite all expectations of living an improved life, some patients became uneasy with their transplanted kidney and why some did not associate their distress with their transplant and/or immunosuppressant medication.

Patients found it difficult to accept their new kidney because of a number of different situations including: the side effects of medication, fear of transplant rejection and feeling obliged to make the most of life because of the “gift” of a transplant. [22-25] It has been reported that concern about potential lifespan of the transplanted kidney may be the biggest stressor immediately after transplant, this fear subsides with time.[8] However, our findings do not reflect this: the majority of our participants, regardless of time since transplant, reported some level of emotional distress.[5] Emotional problems such as depression, anxiety, stress, and concerns about body image are known indicators of poor medication adherence.[3 6] and as discussed, may result in patients wishing they were back on dialysis and in some cases sabotaging their treatment. Gill,[8] Suzuki [26] and Fox [27] found that those receiving a kidney from a living donor had a vested interest in each other’s well-being and were motivated to comply with their medication regimes and enjoyed an improved quality of life. For those receiving a cadaver kidney, thoughts about who their kidney came from were

For peer review only - http://bmjopen.bmj.com/site/about/guidelines.xhtml

forefront in their mind and for some this had a negative effect on their relationship with their kidney – making them feel as though they had a “foreign body” inside them. The emotional distress patients feel may be down to unrealistically high expectations of life post-transplant and there is a need for these expectations to be managed by renal services.[8 23 28]

The majority of participants did not expect to experience distress after a transplant. This may be that patients naively perceive that by having a transplant their quality of life will improve quickly and they will return to a normal life.[29] Consequently, patients are often ill-prepared and feel helpless when trying to cope with their distress.[30] Evidence has shown that improving coping skills,[31] education before and after transplantation,[32] and active information seeking by patients can have a beneficial effect on patients’ medical and psychological problems.[8 23 33] The National Institute for Health and Care Excellence (NICE) advocate keeping patients informed at all stages of treatment and encourage the promotion of self-care and self-management skills.[34] However, HCPs can find it difficult to recognise distress or anxiety in patients and are unsure at which point in the disease trajectory to discuss this with their patients.[35] When a patient is informed, it empowers them to take control of their condition and having control can itself lower the chances of distress.[6] It is therefore important that HCPs provide and share information and discuss all possible outcomes and coping strategies at the appropriate time in the treatment pathway.[3 28 30]

For peer review only - http://bmjopen.bmj.com/site/about/guidelines.xhtml

Not all renal transplant patients will experience distress and not all of those who do will want to receive support. This research has shown that some patients do not wish to talk about their feelings for fear of appearing ungrateful or weak, making it difficult for HCPs to understand, assess and procure appropriate support.[35] Even when patients do want to talk about their feelings, there is "no one size fits all" solution to the provision of support.[36] Different patients have different emotional needs indicating that any support offered to patients should be individualised in order to meet this variance of need.[37]

There were no discernible differences in the depth of data and the length of interview between those conducted face-to-face and those conducted over the telephone. A limitation of this study is that the interview data analysed for this research is a sub-set of a larger set of data. Although the 15 in-depth interviews provided sufficient data to answer the research questions, it is important for future research to explore these areas with a more diverse and carefully stratified sample.

CONCLUSIONS

Our research has highlighted a number of points: firstly, it is important to talk to patients and their families pre-transplant about the possibility of experiencing mild-to-moderate post-transplant distress. Secondly, patients should be encouraged to think about potential coping strategies and finally, transplant patients with mild-to-moderate distress should be able to choose from a variety of support options: peer support; HCPs with augmented skills in detecting and managing distress; access to psychology services.

For peer review only

For peer review only - http://bmjopen.bmj.com/site/about/guidelines.xhtml

Acknowledgements: Francesca Taylor and Elaine Kidney conducted the interviews. Pamela Nayyar provided administrative support for this research. The patient and public involvement group of the CLAHRC West Midlands long-term conditions theme and a renal patient advisory group for their invaluable help and advice. Finally, a huge thank you goes out to all of the participants who gave up their time to talk to us.

Author Contributors: JJ: Conceptualisation (for this sub-study), formal analysis, interpretation of data, methodology, validation, writing (original draft); GC: formal analysis, validation, writing (critical review and editing); SD, & KA: interpretation of data, writing (critical review and editing), JN & JB: Conceptualisation, validation, writing (critical review and editing). All authors have approved the final version.

Funding: This research was funded by the National Institute for Health Research Collaboration for Leadership in Applied Health Research and Care West Midlands (NIHR CLAHRC WM), now recommissioned as NIHR Applied Research Collaboration West Midlands. The views expressed in this publication are those of the author(s) and not necessarily those of the NIHR or the Department of Health and Social Care.

Competing interests: -None

For peer review only - http://bmjopen.bmj.com/site/about/guidelines.xhtml

For peer review only

Ethical approval: The Coventry and Warwickshire Research Ethics Committee granted approval for this and the wider study in October 2015 [Ref 15/WM/0288]. The Research Governance office at each of the participating hospital trusts also gave approval for the study.

Data availability: The research data is confidential. Participants did not give consent to share their data and the ethical requirements of the study do not allow us to share the study data.

For peer review only

REFERENCES

1. UK Renal Registry 21st Annual Report - data to 31/12/2017. Bristol, UK, 2019.
2. Soni RK. Health-related quality of life outcomes in chronic kidney disease. *Curr Opin Nephrol Hypertens*;19(2):153-60. doi: https://doi.org/10.1097/MNH.0b013e328335f939
3. Schell JO, Patel UD, Steinhauser KE, et al. Discussions of the kidney disease trajectory by elderly patients and nephrologists: a qualitative study. *American journal of kidney diseases : the official journal of the National Kidney Foundation* 2012;59(4):495-503. doi: https://doi.org/10.1053/j.ajkd.2011.11.023 [published Online First: 01/04]
4. Murtagh FEM, Addington-Hall J, Higginson IJ. The Prevalence of Symptoms in End-Stage Renal Disease: A Systematic Review. *Advances in Chronic Kidney Disease* 2007;14(1):82-99. doi: https://doi.org/10.1053/j.ackd.2006.10.001
5. Damery S, Brown C, Sein K, et al. The prevalence of mild-to-moderate distress in patients with end-stage renal disease: results from a patient survey using the emotion thermometers in four hospital Trusts in the West Midlands, UK. *BMJ open* 2019;9(5):e027982. doi: https://doi.org/10.1136/bmjopen-2018-027982
6. Cukor D, Rosenthal DS, Jindal RM, et al. Depression is an important contributor to low medication adherence in hemodialyzed patients and transplant recipients. *Kidney Int* 2009;75(11):1223-29. doi: https://doi.org/10.1038/ki.2009.51
7. Combes G, Allen K, Sein K. Evaluation of case study sites for home therapies for people with chronic kidney disease Birmingham: University of Birmingham: West Midlands Central Health Innovation and Education Cluster; 2013 [Available from: http://www.birmingham.ac.uk/research/activity/mds/projects/WMC-HIEC/publications.aspx accessed May 19.
8. Gill P. Stressors and coping mechanisms in live-related renal transplantation. *J Clin Nurs* 2012;21(11-12):1622-31. doi: https://doi.org/10.1111/j.1365-2702.2012.04085.x
9. Kohlsdorf M. Coping Strategies Adopted by Patients With Chronic Kidney Disease in Preparation for Transplant. *Psychol Comm health* 2015;4(1):27-38. doi: https://doi.org/10.5964/pch.v4i1.103 [published Online First: 2015-03-31]

For peer review only - http://bmjopen.bmj.com/site/about/guidelines.xhtml

10. Cukor D, Newville H, Jindal R. Depression and immunosuppressive medication adherence in kidney transplant patients. *General hospital psychiatry* 2008;30(4):386-7. doi: https://doi.org/10.1016/j.genhosppsych.2007.12.003 [published Online First: 2008/07/01]
11. Department of Health, The National Service Framework for Renal Services Part 2: Chronic kidney disease, acute renal failure and end of life care. London: Department of Health 2005.
12. Department of Health, Closing the gap: priorities for essential change in mental health. London: Department of Health 2014
13. Spencer BW, Chilcot J, Farrington K. Still sad after successful renal transplantation: are we failing to recognise depression? An audit of depression screening in renal graft recipients. *Nephron Clinical practice* 2011;117(2):c106-12. doi: https://doi.org/10.1159/000319657 [published Online First: 2010/08/10]
14. Almgren M. The meaning of being in uncertainty after heart transplantation - an unrevealed source to distress. *European Journal of Cardiovascular Nursing* 2017;16(2):167-75. doi: 10.1177/1474515116648240
15. Mauthner OE. Heart transplants: Identity disruption, bodily integrity and interconnectedness. *Health: An Interdisciplinary Journal for the Social Study of Health, Illness & Medicine* 2014;19(6):578-95. doi: 10.1177/1363459314560067
16. Taylor F, Taylor C, Baharani J, et al. Integrating emotional and psychological support into the end-stage renal disease pathway: a protocol for mixed methods research to identify patients' lower-level support needs and how these can most effectively be addressed. *BMC nephrology* 2016;17(1):111. doi: https://doi.org/10.1186/s12882-016-0327-2 [published Online First: 2016/08/04]
17. Mitchell AJ, Baker-Glenn EA, Granger L, et al. Can the Distress Thermometer be improved by additional mood domains? Part I. Initial validation of the Emotion Thermometers tool. *Psycho-Oncology* 2010;19(2):125-33. doi: https://doi.org/10.1002/pon.1523
18. K C, L B. Qualitative interviewing and grounded theory analysis. In: Gubrium JF, Holstein AB, McKinney KD, eds. The SAGE Handbook of interview research: The Complexity of the Craft. California, USA: Sage publications 2012.
19. Braun V, Clarke V. Successful Qualitative Research: a practical guide for beginners. Thousand Oaks, CA: Sage 2013:174-179.
20. Alley S, Jackson SF, Shakya YB. Reflexivity:A Methodological Tool in the Knowledge Translation Process? *Health Promotion Practice* 2015;16(3):426-31. doi: 10.1177/1524839914568344
21. Berger R. Now I see it, now I don't: researcher's position and reflexivity in qualitative research. *Qualitative Research* 2015;15(2):219-34. doi: 10.1177/1468794112468475
22. Achille MA, Ouellette A, Fournier S, et al. Impact of Transplant-Related Stressors and Feelings of Indebtedness on Psychosocial Adjustment Following Kidney Transplantation. *Journal of Clinical Psychology in Medical Settings* 2004;11(1):63-73. doi: 10.1023/B:JOCS.0000016271.10786.ee
23. Chilcot J, Spencer BWJ, Maple H, et al. Depression and Kidney Transplantation. *Transplantation* 2014;97(7):717-21. doi: https://doi.org/10.1097/01.TP.0000438212.72960.ae
24. Shemesh Y, Peles-Bortz A, Peled Y, et al. Feelings of indebtedness and guilt toward donor and immunosuppressive medication adherence among heart transplant (HTx) patients, as assessed in a cross-sectional study with the Basel Assessment of Adherence to Immunosuppressive Medications Scale (BAASIS). *Clinical Transplantation* 2017;31(10):e13053. doi: 10.1111/ctr.13053
25. Poole J, Ward J, Deluca E, et al. Grief and loss for patients before and after heart transplant. *Heart & lung : the journal of critical care* 2016;45(3):193. doi: 10.1016/j.hrtlng.2016.01.006
26. Suzuki A, Kenmochi T, Maruyama M, et al. Changes in quality of life in deceased versus living-donor kidney transplantations. *Transplant Proc* 2012;44(1):287-9. doi: https://doi.org/10.1016/j.transproceed.2011.11.056 [published Online First: 2012/02/09]
27. Fox RC. Through the lenses of biology and sociology: Organ replacement. In: Williams SJ, Birke L, Bendelow GA, eds. Debating Biology: Sociological reflection on health, medicine and society. London: Routledge 2003.

For peer review only - http://bmjopen.bmj.com/site/about/guidelines.xhtml

28. Pinter J, Hanson CS, Craig JC, et al. 'I feel stronger and younger all the time'—perspectives of elderly kidney transplant recipients: thematic synthesis of qualitative research. *Nephrol Dial Transplant* 2016;31(9):1531-40. doi: https://doi.org/10.1093/ndt/gfv463
29. Boaz A, Morgan M. Working to establish 'normality' post-transplant: a qualitative study of kidney transplant patients. *Chronic illness* 2014;10(4):247-58. doi: https://doi.org/10.1177/1742395313504789 [published Online First: 2013/09/28]
30. Cormier NR, Gallo-Cruz SR, Beard RL. Navigating the new, transplanted self: how recipients manage the cognitive risks of organ transplantation. *Sociology of health & illness* 2017;39(8):1496-513. doi: https://doi.org/10.1111/1467-9566.12610 [published Online First: 2017/10/11]
31. Been-Dahmen JMJ, Beck DK, Peeters MAC, et al. Evaluating the feasibility of a nurse-led self-management support intervention for kidney transplant recipients: a pilot study. *BMC nephrology* 2019;20(1):143. doi: https://doi.org/10.1186/s12882-019-1300-7 [published Online First: 2019/04/29]
32. Curcani M, Tan M. the factors affecting the qualilty of life of patients who have undergone kidney transplants *Pakistan Journal of Medicine* 2012;27(5):1092-97.
33. Muehrer RJ, Becker BN. Life after transplantation: new transitions in quality of life and psychological distress. *Semin Dial* 2005;18(2):124-31. doi: https://doi.org/10.1111/j.1525-139X.2005.18214.x [published Online First: 2005/03/18]
34. National Institute for Health and Clinical Excellence, CG138 Patient experience in NHS services: improving the experience of care for people using adult NHS services Manchester: National Institute for Health and Clinical Excellence 2012 [Available from: http://guidance.nice.org.uk/cg138 accessed July 2019.
35. Damery S, Sein K, Combes G. The prevalence, experience and management of mild to moderate distress in patients with end stage renal disease: results from a multi-centre, mixed methods research study: NIHR CLAHRC-WM, University of Birmingham, 2019.
36. Been-Dahmen JMJ, Grijpma JW, Ista E, et al. Self-management challenges and support needs among kidney transplant recipients: A qualitative study. *Journal of advanced nursing* 2018;74(10):2393-405. doi: https://doi.org/10.1111/jan.13730 [published Online First: 2018/06/06]
37. Grijpma JW, Tielen M, van Staa AL, et al. Kidney transplant patients' attitudes towards self-management support: A Q-methodological study. *Patient Educ Couns* 2016;99(5):836-43. doi: https://doi.org/10.1016/j.pec.2015.11.018 [published Online First: 2015/12/20]

For peer review only

For peer review only - http://bmjopen.bmj.com/site/about/guidelines.xhtml

COREQ (COnsolidated criteria for REporting Qualitative research) Checklist

A checklist of items that should be included in reports of qualitative research. You must report the page number in your manuscript where you consider each of the items listed in this checklist. If you have not included this information, either revise your manuscript accordingly before submitting or note N/A.

Topic	Item No.	Guide Questions/Description	Reported on Page No.
**Domain 1: Research team and reflexivity**			
Personal characteristics			
Interviewer/facilitator	1	Which author/s conducted the interview or focus group?	6
Credentials	2	What were the researcher's credentials? E.g. PhD, MD	6
Occupation	3	What was their occupation at the time of the study?	6
Gender	4	Was the researcher male or female?	6
Experience and training	5	What experience or training did the researcher have?	6
Relationship with participants			
Relationship established	6	Was a relationship established prior to study commencement?	6
Participant knowledge of the interviewer	7	What did the participants know about the researcher? e.g. personal goals, reasons for doing the research	6
Interviewer characteristics	8	What characteristics were reported about the inter viewer/facilitator? e.g. Bias, assumptions, reasons and interests in the research topic	6
**Domain 2: Study design**			
Theoretical framework			
Methodological orientation and Theory	9	What methodological orientation was stated to underpin the study? e.g. grounded theory, discourse analysis, ethnography, phenomenology, content analysis	Referred to pr[illegible]
Participant selection			
Sampling	10	How were participants selected? e.g. purposive, convenience, consecutive, snowball	6
Method of approach	11	How were participants approached? e.g. face-to-face, telephone, mail, email	6
Sample size	12	How many participants were in the study?	8
Non-participation	13	How many people refused to participate or dropped out? Reasons?	Data not ava[illegible]
Setting			
Setting of data collection	14	Where was the data collected? e.g. home, clinic, workplace	7
Presence of non-participants	15	Was anyone else present besides the participants and researchers?	Data not availa[illegible]
Description of sample	16	What are the important characteristics of the sample? e.g. demographic data, date	8
Data collection			
Interview guide	17	Were questions, prompts, guides provided by the authors? Was it pilot tested?	7
Repeat interviews	18	Were repeat inter views carried out? If yes, how many?	N/A
Audio/visual recording	19	Did the research use audio or visual recording to collect the data?	7
Field notes	20	Were field notes made during and/or after the inter view or focus group?	7
Duration	21	What was the duration of the inter views or focus group?	7
Data saturation	22	Was data saturation discussed?	19
Transcripts returned	23	Were transcripts returned to participants for comment and/or	7

For peer review only

For peer review only - http://bmjopen.bmj.com/site/about/guidelines.xhtml

Topic	Item No.	Guide Questions/Description	Reported on Page No.
		correction?	
**Domain 3: analysis and findings**			
Data analysis			
Number of data coders	24	How many data coders coded the data?	7
Description of the coding tree	25	Did authors provide a description of the coding tree?	9
Derivation of themes	26	Were themes identified in advance or derived from the data?	7
Software	27	What software, if applicable, was used to manage the data?	7
Participant checking	28	Did participants provide feedback on the findings?	7
Reporting			
Quotations presented	29	Were participant quotations presented to illustrate the themes/findings? Was each quotation identified? e.g. participant number	9-16
Data and findings consistent	30	Was there consistency between the data presented and the findings?	9-16
Clarity of major themes	31	Were major themes clearly presented in the findings?	9-16
Clarity of minor themes	32	Is there a description of diverse cases or discussion of minor themes?	10

Developed from: Tong A, Sainsbury P, Craig J. Consolidated criteria for reporting qualitative research (COREQ): a 32-item checklist for interviews and focus groups. *International Journal for Quality in Health Care*. 2007. Volume 19, Number 6: pp. 349 – 357

Once you have completed this checklist, please save a copy and upload it as part of your submission. DO NOT include this checklist as part of the main manuscript document. It must be uploaded as a separate file.

For peer review only

BMJ Open

"You've got a foreign body in there": Renal transplantation, unexpected mild-to-moderate distress and patients' support needs: a qualitative study

Journal:	*BMJ Open*
Manuscript ID	bmjopen-2019-035627.R2
Article Type:	Original research
Date Submitted by the Author:	20-Jan-2020
Complete List of Authors:	Jones, Janet ; University of Birmingham, Institute of Applied Health Research<br>Damery, Sarah; University of Birmingham, Institute of Applied Health Research<br>Allen, Kerry; University of Birmingham, Health Services Management Centre<br>Nicholas, Johann; Shrewsbury and Telford Hospital NHS Trust, Renal Unit<br>Baharani, Jyoti; Heart of England NHS Foundation Trust, Renal Medicine<br>Combes, Gill; University of Birmingham, Institute of Applied Health Research
**Primary Subject Heading**:	Renal medicine
Secondary Subject Heading:	Qualitative research
Keywords:	Renal transplantation < NEPHROLOGY, Anxiety disorders < PSYCHIATRY, QUALITATIVE RESEARCH

SCHOLARONE™
Manuscripts

I, the Submitting Author has the right to grant and does grant on behalf of all authors of the Work (as defined in the below author licence), an exclusive licence and/or a non-exclusive licence for contributions from authors who are: i) UK Crown employees; ii) where BMJ has agreed a CC-BY licence shall apply, and/or iii) in accordance with the terms applicable for US Federal Government officers or employees acting as part of their official duties; on a worldwide, perpetual, irrevocable, royalty-free basis to BMJ Publishing Group Ltd ("BMJ") its licensees and where the relevant Journal is co-owned by BMJ to the co-owners of the Journal, to publish the Work in this journal and any other BMJ products and to exploit all rights, as set out in our licence.

The Submitting Author accepts and understands that any supply made under these terms is made by BMJ to the Submitting Author unless you are acting as an employee on behalf of your employer or a postgraduate student of an affiliated institution which is paying any applicable article publishing charge ("APC") for Open Access articles. Where the Submitting Author wishes to make the Work available on an Open Access basis (and intends to pay the relevant APC), the terms of reuse of such Open Access shall be governed by a Creative Commons licence – details of these licences and which Creative Commons licence will apply to this Work are set out in our licence referred to above.

Other than as permitted in any relevant BMJ Author's Self Archiving Policies, I confirm this Work has not been accepted for publication elsewhere, is not being considered for publication elsewhere and does not duplicate material already published. I confirm all authors consent to publication of this Work and authorise the granting of this licence.

For peer review only

"You've got a foreign body in there": Renal transplantation, unexpected mild-to-moderate distress and patients' support needs: a qualitative study

Short title: Unexpected distress in renal transplant patients

Janet Jones*[1], Sarah Damery[1], Kerry Allen[2], Johann Nicholas[3^], Jyoti Baharani[4], and Gill Combes[1],

[1]Institute of Applied Health Research, University of Birmingham, Birmingham UK.

[2]Health Services Management Centre, University of Birmingham, Birmingham, UK.

[3]Renal Unit, New Cross Hospital, Royal Wolverhampton NHS Trust, Wolverhampton,

[4]Renal Unit, Birmingham Heartlands Hospital, University Hospital Birmingham NHS Foundation Trust, Birmingham, UK.

*Corresponding author at: Institute of Applied Health Research, University of Birmingham, Edgbaston, Birmingham B15 2TT, UK.

E mail address: j.e.jones@bham.ac.uk, Tel number: 0121 414 7661.

^ Current affiliation: Renal Unit, Royal Shrewsbury Hospital, Shrewsbury, UK

Word count: 3555

For peer review only

ABSTRACT

Objective: To explore why transplant patients experience unexpected mild-moderate distress and what support they may need.

Design: Qualitative study using individual in-depth interviews.

Setting: Four NHS Trusts in the Midlands, UK.

Participants: Fifteen renal transplant patients meeting the criteria for mild to moderate distress from their responses to emotion thermometers.

Main outcome measures: identification of the reasons for distress and support options acceptable to renal transplant patients.

Results: Three themes were interpreted from the data: 'I'm living with a "foreign body" inside me', 'why am I distressed?' and, 'different patients want different support'. Following their transplant, participants felt that they should be happy and content, but this was often not the case. They described a range of feelings about their transplant, such as: uncertainty about the lifespan of their new kidney, fear of transplant failure or fear of the donor having health conditions that may transfer to them. A few experienced survivors' guilt when others they had met at the dialysis unit had not received a transplant or because someone had died to enable them to receive the transplant. No longer having regular contact with the renal unit made participants feel isolated. Some participants did not initially attribute the source of their distress to their transplant. Participants' preferred support for their distress and their preferences about who should deliver it varied from peer support to seeing a psychologist.

For peer review only

Conclusion: Raising the issue of post-transplant mild-to-moderate distress with patients and encouraging them to think about and plan coping strategies pre-transplant may prove beneficial for the patient and healthcare provider. Patients should be able to choose from a variety of support options.

ARTICLE SUMMARY

Strengths and limitations of this study

- This is the first study designed to explore the issue of unexpected mild-to-moderate distress in renal transplant patients.
- The Interviews were a sub-set of data from a larger study.
- To reduce bias on the basis of the services offered to renal patients multiple sites with different organisation and delivery of services were selected.
- Participants were diverse thus the findings are more representative of the wider group of renal transplant patients.

For peer review only

For peer review only - http://bmjopen.bmj.com/site/about/guidelines.xhtml

INTRODUCTION

At the end of 2017, 63,162 adults were receiving renal replacement therapy in the UK [1], and in the same year 3,462 transplants were performed.[1] Patients with end stage renal disease (ESRD) can experience distress and distress is associated with lower quality of life and greater treatment burden.[2] The level of emotional distress increases as the health of a patient with ESRD declines [3] and the prevalence of depression and anxiety in patients with ESRD is approximately four times higher than in the general adult population.[4] Damery et al 2019 [5] reported that more than a third of renal dialysis patients suffer emotional distress. If suffering from mild-to-moderate distress – the unpleasant feelings or emotions that may interfere with patients' ability to cope with their kidney transplant, its physical symptoms and its treatments - patients may withdraw from treatment, be non-compliant with medication and diet [6] or be reluctant to engage in pre-renal replacement therapy (RRT) education and support.[7]

Although not a cure, for patients with ESRD, receiving a transplant provides the best chance of improving their quality of life and removing the burden of undergoing long-term dialysis treatment.[8 9] Nevertheless, research has shown that although receiving a transplant is the main goal for ESRD patients who are eligible for transplantation, they may continue to experience distress. For example, fear of the transplant failing can affect patient distress levels.[8] There is also evidence that patients may experience ongoing physical symptoms such as fatigue after receiving a transplant, and that there is substantial burden associated with taking regular immunosuppressant medication to lower the likelihood of graft rejection.[10] UK health

For peer review only - http://bmjopen.bmj.com/site/about/guidelines.xhtml

policies highlight the importance of addressing the emotional and psychological needs of renal transplant patients [11] and the Department of Health and NHS England advocate treating mental health on an equal footing to that of physical health by incorporating it into care pathways.[12] Nevertheless, evidence suggests that distress, coping, and adjustment in transplant patients largely go undiagnosed or ignored and remain untreated.[13] Currently, there is little evidence on mild-to-moderate distress in transplant patients and further research is required in order to understand the psychological and emotional effects of a transplant.[9 14 15]

As an element of a larger mixed-methods study with patients and staff,[16] this paper reports the findings from a qualitative study with renal transplant patients. The aims were to explore why transplant patients experience distress and what support they may need.

METHODS

The detailed methods for the study are in the published protocol.[16] In brief, participants were recruited from four National Health Service (NHS) hospitals in the West Midlands, UK. The chosen sites provided maximum diversity in patient demographics, catchment size, urban and rural locations plus the organisation of psychological support services and were categorised according to the size of the catchment area. Table 1.

For peer review only - http://bmjopen.bmj.com/site/about/guidelines.xhtml

Table 1. Summary of recruitment sites

Site	Size	Catchment area	On-site renal psychologist
1	Small	Urban inner city with sizable Black, Asian and minority ethnic (BAME) population	No
2	Large	Urban inner city with sizable BAME population	Yes
3	Medium	Urban with surrounding rural districts majority white population	No
4	Large	Urban with surrounding rural districts majority white population	Yes

To be eligible for recruitment to the qualitative study, patients had to be categorised as having mild-to-moderate distress based on their responses to the distress thermometer [17] included in the wider study questionnaire. Participants indicated on the questionnaire whether or not they would be willing to take part in an interview and provided their preferred contact details. A consent form and participant information sheet explaining the purpose of the interview study and what participation would involve were sent to those expressing interest. The following week those meeting the purposive sampling criteria (age, sex and ethnicity) were contacted by a researcher (FT) to confirm participation and arrange a date and time for the interview. If participants had changed their mind they were able to withdraw from this part of the wider study. As well as assuring patient confidentiality, the patient information sheet provided contact details of appropriate clinical staff the participants could contact if they felt distressed or upset and would like support. Contacting the participants to answer their questions, to arrange the interview and to

For peer review only

For peer review only - http://bmjopen.bmj.com/site/about/guidelines.xhtml

remind them a few days beforehand helped to build up a rapport with each participant prior to their interview.

Patient interviews took place between March 2016 and May 2017 and were conducted by two experienced qualitative researchers (FT, EK) (both identifying as female and educated to masters level) employed by the University of Birmingham. Neither researcher had experience (personally or professionally) of the topic area and none of the participants knew the researchers. Interviews were either face-to-face (at a location chosen by the participant, either a quiet room at the hospital or at the patients home) or over the telephone and lasted between 30 - 60 minutes. The interviews were in-depth and semi-structured allowing the exploration of key issues without being overly prescriptive about content and direction. All participants provided signed written informed consent prior to their interview. Patient advisors, renal clinicians and the current literature helped with the design of the topic guide, see box 1 for the areas covered by the topic guide. Prior to the start of the interview participants were advised to let the researcher know if they needed to take a break during the interview or if they no longer wished to carry on. Although included in the participant information sheet participants were reminded that taking part in an interview would not impact on the care they receive. At the end of each interview participants were asked if they had any further comments on the topics covered or whether there were any important areas they felt had not been discussed. Both researchers made field notes following each interview. Interviews were audio-recorded and transcribed verbatim by a professional transcribing service. Transcripts were checked against the recordings for accuracy. Participants did not have the opportunity to review their interview transcript.

For peer review only - http://bmjopen.bmj.com/site/about/guidelines.xhtml

a) Experience of emotional difficulties and needs linked to their illness and/or treatment, when and for how long.
b) Language used around emotional difficulties and needs, and its meaning.
c) Whether and how emotional needs have been recognised and supported by renal staff, when and by whom.
d) What, if any, support used, when and why.
e) Likes and dislikes of support used.
f) Support would have liked/would want in future, when, and from whom.
g) Key elements would want included in an emotional support intervention

Box 1. Areas covered by the topic guide

Analysis combined aspects of grounded theory [18] and thematic analysis.[19] Interviews were initially analysed inductively using the open coding and constant comparison aspects of grounded theory. The initial coding framework was developed by JJ and CG and was appropriately refined following comparison and discussion. Transcripts were coded using NVivo 11. For data that did not fit existing themes, new codes were developed or existing ones revised until all data were coded by theme. Following the completion of data collection the research team met and agreed that in order understand participants' experiences and to help inform future practice a generic pragmatic hybrid approach to analysis was appropriate [20 21]. The research team also discussed the role of reflexivity and how our personal views and experiences may influence our interpretation of the data. [22 23]

Patient and public involvement

For peer review only - http://bmjopen.bmj.com/site/about/guidelines.xhtml

The patient and public involvement group of the NIHR CLAHRC West Midlands long-term conditions theme and a renal patient advisory group provided advice on the design of the study, the data collection tools and the selection of outcomes. All participants received a summary of the study findings.

RESULTS

Fifteen renal transplant patients aged between 30 and ≥70 years were recruited across the four sites, see tables 2 and 3.

Table 2. Summary of participant characteristics

		N (%)
**Sex (participant self-identified)**	Male	7 (47)
	Female	8 (53)
**Age**	30-39	3 (20)
	40-49	2 (13)
	50-59	4 (27)
	60-69	4 (27)
	≥70	2 (13)
***Ethnicity (participant self-identified)**	White	9 (60)
	Indian	4 (27)
	Caribbean	2 (13)
**Length of time on dialysis prior to transplant**	No dialysis	1 (7)
	0 – 3 years	5 (33)
	4 – 9 years	0 (0)
	≥ 10 years	3 (20)
	Unknown	6 (40)

*According to the Office of National Statistics ethnicity groupings 2015

Table 3. Individual participant characteristics

ID	Age	Time on dialysis prior to transplant
P129	40-49	No dialysis
P267	60-69	Not stated

For peer review only - http://bmjopen.bmj.com/site/about/guidelines.xhtml

P384	50-59	16 ½ years
P413	30-39	Not stated
P494	50-59	11 years
P687	30-39	Not stated
P726	50-59	10 years
P781	≥70	11 months
P197	40-49	1 year
P225	≤70	3 years
P369	60-69	Not stated
P389	50-59	3 years
P401	30-39	Not stated
P1028	60-69	6 months
P1141	60-69	Not stated

Three overarching themes were interpreted from the data. See table 4 for a summary of the themes and sub-themes:

Table 4. Themes and sub-themes

Theme	Sub-themes
I'm living with a "foreign body" inside me	Fear/feelings about the kidney itself
	Survivors' guilt
	Feelings of isolation
	Impacts of medication
Why am I distressed?	Expectations of living with a transplant
	Coping with distress
	Lack of information about transplants and support
Different patients want different types of support	

I'm living with a "foreign body" inside me

Fear/feelings about the kidney itself

Living with a transplanted kidney and its associated treatments can evoke many different feelings and difficulties for patients. For many there is the fear and uncertainty of how long the transplant will last: participants talked about their

For peer review only - http://bmjopen.bmj.com/site/about/guidelines.xhtml

transplant having a finite life and how this knowledge made them worry about what would happen in the future. Three participants had experienced a failed transplant and others understood the status of their transplant could change at any time:

> *"A lot of people I knew before when I was on dialysis who had transplants, they had rejections and all sorts of things. So that's passing through your mind all the while".* (P225).

Among some patients, their fear had escalated the longer they had been with a transplant, particularly after their transplant exceeded the average lifespan. Some patients thought the longevity of a transplanted kidney was something of a lottery, which was percevied to be outside of their control and could therefore cause distress. A few patients seemed emotionally affected by feeling they had a "foreign body" inside them (M225). One male patient talked about the possible consequences for his character of having received a female kidney. Another was anxious that his transplanted kidney might have come from someone with other health problems:

> *"When I got it I started to get awful thoughts at night. 'How did this person die, how old were they, did they have anything else, could they possibly have been HIV positive? Could there have been any other things that were underlying that may come forward later on?' and I still get those sort of thoughts at the moment".* (P1141).

Survivors' guilt

For peer review only - http://bmjopen.bmj.com/site/about/guidelines.xhtml

Although a transplant is the gold standard treatment for ESRD, some participants mentioned feeling guilty about being distressed and some worried this may make them appear ungrateful. They felt they should be happy because they were lucky to receive a transplant: "*but you, at the same time you've got no reason to feel like that".* (M389). This guilt and the fear of appearing ungrateful prevented some from seeking help when they needed it and stopped them from moving forward with their lives. Some participants worried about the donor, what happened to them and the family they left behind and others had survivors' guilt because they had received their transplant before others who had been waiting longer:

> *"When I had my transplant I felt, I suppose what you'd call it is like in a sense survivors guilt…..And because I had my transplant so quickly I just felt this huge amount of guilt because I thought all those patients that I had met at* [hospital] *and they've been on the list for such a long time, and I thought 'what's the difference between me and them?'".* (P413).

Feelings of isolation

There was a perception amongst several of the participants that after receiving a transplant they were no longer part of the renal unit. They talked about feeling 'cut-off' and 'abandoned' with minimal contact. One patient explained how there had been no contact with his renal unit for several years following his transplant. Patients that had transitioned from in-centre haemodialysis (HD) seemed to feel this loss acutely - they missed the supportive relationship of staff and patients in the dialysis unit:

For peer review only - http://bmjopen.bmj.com/site/about/guidelines.xhtml

"Once you're transplanted, you don't really have anything to do as such with the renal unit……the only time I actually only ever was involved with the renal unit was when I was on dialysis. So that sort of like support was gone". (P726).

Some patients experienced feelings of isolation because of the lack of understanding about their disease and its treatments among family, friends and society in general. They believed only fellow transplant patients understood their feelings:

"It helped the fact talking to a stranger about it. You know, the wife couldn't understand why I was like it and that, but obviously the stranger could because she knew the experience of it all". (P369).

Impacts of medication

Feeling down and finding their situation difficult to deal with was widespread amongst the participants. These feelings were often present when participants were dealing with the physical side effects of the anti-rejection medications such as, weight gain, puffy appearance and excessive hair growth. Other peoples' reactions to the physical changes were often difficult to deal with:

"You know like the new face and excessive hair ….. and I found that I think the most difficult for me was that change and unfortunately when I went back to school I had a bit of , a bit of bullying went on, because of physically changing so much from what I was previously as well". (P687).

For peer review only - http://bmjopen.bmj.com/site/about/guidelines.xhtml

Participants explained how when they were on dialysis they felt in control of their body, their treatment and their lives but following transplant many felt they no longer had command of their body or life in general and yearned to take back control. Such as, not wanting to leave the house because of lack of bladder control. For the following patient the feeling of helplessness ended with her sabotaging her treatment:

> *"So the reason my kidney failed was because I felt like I didn't have much control, so I kind of stopped taking my pills, my immunosuppressant. I stopped taking them for a while".* (P413).

Why am I distressed?

Expectations of living with a transplant

Following their transplant, participants did not expect to experience distress because they regarded a transplant as the best treatment option for an improved quality of life. Some patients had waited many years for the opportunity to receive the 'gift' of a transplant and had been optimistic:

> *"I'm coming at it first time with an expectation that once you get a transplant and you start feeling better again, life is rosy, life can get back to normal".* (P389).

Whilst experiencing distress, many participants did not initially relate it to their transplant. The data suggest that the reasons for this were two-fold. Firstly, following

For peer review only

For peer review only - http://bmjopen.bmj.com/site/about/guidelines.xhtml

a transplant there is an expectation from family, friends, clinicians and society in general that life will return to 'normal' and be 'wonderful' again.

> *"I never did link it to – there was something in your paperwork that I had. I thought I've never associated it with the kidney operation".* (P369).

Secondly, renal staff did not forewarn the participants they might experience distress after receiving a transplant. Some only made the connection after agreeing to take part in this research study:

> *But being in the hospital and then having all sorts of side effects which is effecting me emotionally now. And that things could have been told to me before, you know, I would have prepped myself up. Some of those things could have been addressed before. It could have been helpful to have been told beforehand. (P129).*

Coping with distress

Renal disease and its lifecycle can make it hard for patients to be positive about their transplant. Several participants explained how it was difficult to cope with their emotions, to move on with their lives and how they perceived family and friends, in particular, were defining them by their health condition:

> *"(Sighs) Ah, again I suppose it comes back to the fact that I don't want to like, yes like I know I've got an illness, but I don't want to be*

For peer review only - http://bmjopen.bmj.com/site/about/guidelines.xhtml

that's who I am like. You know that's what I'm all about sort of thing". (P726).

The majority of participants developed ways of accepting post-transplant life and found ways to cope with their distress. For some, distractions in the form of hobbies and pastimes such as gardening or reading helped them to adjust and for others it was important to maintain a positive outlook about their transplant:

"I've always had this positive outlook on the transplant and that." (P225).

Lack of information about transplants and support

Many participants wished the renal team had explained to them the possibility of experiencing post-transplant distress and lamented the lack of information about this and the lack of available support. Many felt that "forewarned is forearmed" and were upset they missed the opportunity to plan, in advance, coping strategies. This lack of information provision was associated with a lack of continuity of care, and participants felt that staff regarded transplant patients as a lower priority compared to those on dialysis:

"Time is precious, resources are scarce, I suspect that probably it never feels as though it's a big priority". (P389).

Different patients want different types of support

For peer review only - http://bmjopen.bmj.com/site/about/guidelines.xhtml
For peer review only

There were, however, diverse views amongst the participants about the types of support they would like to receive, and who should deliver it. For example, one participant described how she thought talking to other patients might have provided the support she sought.

> *"So with other patients in your situation, so that you could liaise with each other to see what new life is all about".* (P494).

There were mixed views about the role of healthcare professionals (HCPs), with some suggesting that HCPs should be more proactive in identifying distress in renal transplant patients and directing them to appropriate sources of help and support. Whereas others did not see this as the role of the renal team – they are there to provide medical assistance and advice not psychological and emotional support. Many suggested that specialist psychological services should be available as in integral part of care for renal transplant patients:

> *"I think you need psychologists as part of the renal team, a psychologist with renal expertise".* (P781).

However, because of the sensitive and personal nature of distress or for fear of showing weakness there were those who were reluctant to talk about their distress to anyone, making it difficult for HCPs to assess the support needs of these patients:

For peer review only - http://bmjopen.bmj.com/site/about/guidelines.xhtml

"You know, it might have been something to do with medication – I'm just guessing. But I should have told them but as I say, I mean it's not the sort of thing I tell people" (P369).

DISCUSSION

This research has highlighted the complex relationship that renal transplant patients have with their new kidney, their largely unanticipated experience of their distress and the diverse opinions on the types of support they would like to help them through their distress. Current research has shown that the prevalence of distress in renal transplant patients is 25% and although this is lower than the 33% of dialysis patients, it is still substantial and shows that many transplant patients experience ongoing issues.[5] There is a range of reasons why despite all expectations of living an improved life, some patients became uneasy with their transplanted kidney and why some did not associate their distress with their transplant and/or immunosuppressant medication.

Patients found it difficult to accept their new kidney because of a number of different situations including: the side effects of medication, fear of transplant rejection and feeling obliged to make the most of life because of the "gift" of a transplant. [24-27] It has been reported that concern about potential lifespan of the transplanted kidney may be the biggest stressor immediately after transplant, this fear subsides with time.[8] However, our findings do not reflect this: the majority of our participants, regardless of time since transplant, reported some level of emotional distress.[5] Emotional problems such as depression, anxiety, stress, and concerns about body

For peer review only - http://bmjopen.bmj.com/site/about/guidelines.xhtml

image are known indicators of poor medication adherence.[3 6] and as discussed, may result in patients wishing they were back on dialysis and in some cases sabotaging their treatment. Gill,[8] Suzuki [28] and Fox [29] found that those receiving a kidney from a living donor had a vested interest in each other's well-being and were motivated to comply with their medication regimes and enjoyed an improved quality of life. For those receiving a cadaver kidney, thoughts about who their kidney came from were forefront in their mind and for some this had a negative effect on their relationship with their kidney – making them feel as though they had a "foreign body" inside them. The emotional distress patients feel may be down to unrealistically high expectations of life post-transplant and there is a need for these expectations to be managed by renal services.[8 25 30]

The majority of participants did not expect to experience distress after a transplant. This may be that patients naively perceive that by having a transplant their quality of life will improve quickly and they will return to a normal life.[31] Consequently, patients are often ill-prepared and feel helpless when trying to cope with their distress.[32] Evidence has shown that improving coping skills,[33] education before and after transplantation,[34] and active information seeking by patients can have a beneficial effect on patients' medical and psychological problems.[8 25 35] The National Institute for Health and Care Excellence (NICE) advocate keeping patients informed at all stages of treatment and encourage the promotion of self-care and self-management skills.[36] However, HCPs can find it difficult to recognise distress or anxiety in patients and are unsure at which point in the disease trajectory to discuss this with their patients.[37] When a patient is informed, it empowers them to take control of their condition and having control can itself lower the chances of distress.[6] It is therefore

For peer review only - http://bmjopen.bmj.com/site/about/guidelines.xhtml

important that HCPs provide and share information and discuss all possible outcomes and coping strategies at the appropriate time in the treatment pathway.[3 30 32]

Not all renal transplant patients will experience distress and not all of those who do will want to receive support. This research has shown that some patients do not wish to talk about their feelings for fear of appearing ungrateful or weak, making it difficult for HCPs to understand, assess and procure appropriate support.[37] Even when patients do want to talk about their feelings, there is “no one size fits all” solution to the provision of support.[38] Different patients have different emotional needs indicating that any support offered to patients should be individualised in order to meet this variance of need.[39]

There were no discernible differences in the depth of data and the length of interview between those conducted face-to-face and those conducted over the telephone. A limitation of this study is that the interview data analysed for this research is a sub-set of a larger set of data. Although the 15 in-depth interviews provided sufficient data to answer the research questions and were more representative of the wider group of transplant patients, it is important for future research to explore these areas with a more diverse and carefully stratified sample.

CONCLUSIONS

For peer review only - http://bmjopen.bmj.com/site/about/guidelines.xhtml

Our research has highlighted a number of points: firstly, it is important to talk to patients and their families pre-transplant about the possibility of experiencing mild-to-moderate post-transplant distress. Secondly, patients should be encouraged to think about potential coping strategies and finally, transplant patients with mild-to-moderate distress should be able to choose from a variety of support options: peer support; HCPs with augmented skills in detecting and managing distress; access to psychology services.

Acknowledgements: Francesca Taylor and Elaine Kidney conducted the interviews. Pamela Nayyar provided administrative support for this research. The patient and public involvement group of the CLAHRC West Midlands long-term conditions theme and a renal patient advisory group for their invaluable help and advice. Finally, a huge thank you goes out to all of the participants who gave up their time to talk to us.

Author Contributors: JJ: Conceptualisation (for this sub-study), formal analysis, interpretation of data, methodology, validation, writing (original draft); GC: formal analysis, validation, writing (critical review and editing); SD, & KA: interpretation of data, writing (critical review and editing), JN & JB: Conceptualisation, validation, writing (critical review and editing). All authors have approved the final version.

Funding: This research was funded by the National Institute for Health Research Collaboration for Leadership in Applied Health Research and Care West Midlands (NIHR CLAHRC WM), now recommissioned as NIHR Applied Research

For peer review only - http://bmjopen.bmj.com/site/about/guidelines.xhtml

Collaboration West Midlands. The views expressed in this publication are those of the author(s) and not necessarily those of the NIHR or the Department of Health and Social Care.

Competing interests: -None

Ethical approval: The Coventry and Warwickshire Research Ethics Committee granted approval for this and the wider study in October 2015 [Ref 15/WM/0288]. The Research Governance office at each of the participating hospital trusts also gave approval for the study.

Data availability: The research data is confidential. Participants did not give consent to share their data and the ethical requirements of the study do not allow us to share the study data.

For peer review only

REFERENCES

1. UK Renal Registry 21st Annual Report - data to 31/12/2017. Bristol, UK, 2019.
2. Soni RK. Health-related quality of life outcomes in chronic kidney disease. *Curr Opin Nephrol Hypertens*;19(2):153-60. doi: https://doi.org/10.1097/MNH.0b013e328335f939
3. Schell JO, Patel UD, Steinhauser KE, et al. Discussions of the kidney disease trajectory by elderly patients and nephrologists: a qualitative study. *American journal of kidney diseases : the official journal of the National Kidney Foundation* 2012;59(4):495-503. doi: https://doi.org/10.1053/j.ajkd.2011.11.023 [published Online First: 01/04]
4. Murtagh FEM, Addington-Hall J, Higginson IJ. The Prevalence of Symptoms in End-Stage Renal Disease: A Systematic Review. *Advances in Chronic Kidney Disease* 2007;14(1):82-99. doi: https://doi.org/10.1053/j.ackd.2006.10.001
5. Damery S, Brown C, Sein K, et al. The prevalence of mild-to-moderate distress in patients with end-stage renal disease: results from a patient survey using the emotion thermometers in four

For peer review only - http://bmjopen.bmj.com/site/about/guidelines.xhtml

hospital Trusts in the West Midlands, UK. *BMJ open* 2019;9(5):e027982. doi: https://doi.org/10.1136/bmjopen-2018-027982
6. Cukor D, Rosenthal DS, Jindal RM, et al. Depression is an important contributor to low medication adherence in hemodialyzed patients and transplant recipients. *Kidney Int* 2009;75(11):1223-29. doi: https://doi.org/10.1038/ki.2009.51
7. Combes G, Allen K, Sein K. Evaluation of case study sites for home therapies for people with chronic kidney disease Birmingham: University of Birmingham: West Midlands Central Health Innovation and Education Cluster; 2013 [Available from: http://www.birmingham.ac.uk/research/activity/mds/projects/WMC-HIEC/publications.aspx accessed May 19.
8. Gill P. Stressors and coping mechanisms in live-related renal transplantation. *J Clin Nurs* 2012;21(11-12):1622-31. doi: https://doi.org/10.1111/j.1365-2702.2012.04085.x
9. Kohlsdorf M. Coping Strategies Adopted by Patients With Chronic Kidney Disease in Preparation for Transplant. *Psychol Comm health* 2015;4(1):27-38. doi: https://doi.org/10.5964/pch.v4i1.103 [published Online First: 2015-03-31]
10. Cukor D, Newville H, Jindal R. Depression and immunosuppressive medication adherence in kidney transplant patients. *General hospital psychiatry* 2008;30(4):386-7. doi: https://doi.org/10.1016/j.genhosppsych.2007.12.003 [published Online First: 2008/07/01]
11. Department of Health, The National Service Framework for Renal Services Part 2: Chronic kidney disease, acute renal failure and end of life care. London: Department of Health 2005.
12. Department of Health, Closing the gap: priorities for essential change in mental health. London: Department of Health 2014
13. Spencer BW, Chilcot J, Farrington K. Still sad after successful renal transplantation: are we failing to recognise depression? An audit of depression screening in renal graft recipients. *Nephron Clinical practice* 2011;117(2):c106-12. doi: https://doi.org/10.1159/000319657 [published Online First: 2010/08/10]
14. Almgren M. The meaning of being in uncertainty after heart transplantation - an unrevealed source to distress. *European Journal of Cardiovascular Nursing* 2017;16(2):167-75. doi: 10.1177/1474515116648240
15. Mauthner OE. Heart transplants: Identity disruption, bodily integrity and interconnectedness. *Health: An Interdisciplinary Journal for the Social Study of Health, Illness & Medicine* 2014;19(6):578-95. doi: 10.1177/1363459314560067
16. Taylor F, Taylor C, Baharani J, et al. Integrating emotional and psychological support into the end-stage renal disease pathway: a protocol for mixed methods research to identify patients' lower-level support needs and how these can most effectively be addressed. *BMC Nephrol* 2016;17(1):111. doi: https://doi.org/10.1186/s12882-016-0327-2 [published Online First: 2016/08/04]
17. Mitchell AJ, Baker-Glenn EA, Granger L, et al. Can the Distress Thermometer be improved by additional mood domains? Part I. Initial validation of the Emotion Thermometers tool. *Psycho-Oncology* 2010;19(2):125-33. doi: https://doi.org/10.1002/pon.1523
18. K C, L B. Qualitative interviewing and grounded theory analysis. In: Gubrium JF, Holstein AB, McKinney KD, eds. The SAGE Handbook of interview research: The Complexity of the Craft. California, USA: Sage publications 2012.
19. Braun V, Clarke V. Successful Qualitative Research: a practical guide for beginners. Thousand Oaks, CA: Sage 2013:174-179.
20. Caelli K, Ray L, Mill J. 'Clear as Mud': Toward greater clarity in generic qualitative research. *Int J Qual Methods* 2003;2(2):1-24.
21. Cooper S, Endacott R. Generic qualitative research: a design for qualitative research in emergency care? *Emergency Medicine Journal : EMJ* 2007;24(12):816-19. doi: 10.1136/emj.2007.050641
22. Alley S, Jackson SF, Shakya YB. Reflexivity:A Methodological Tool in the Knowledge Translation Process? *Health Promotion Practice* 2015;16(3):426-31. doi: 10.1177/1524839914568344

For peer review only - http://bmjopen.bmj.com/site/about/guidelines.xhtml

23. Berger R. Now I see it, now I don't: researcher's position and reflexivity in qualitative research. *Qualitative Research* 2015;15(2):219-34. doi: 10.1177/1468794112468475
24. Achille MA, Ouellette A, Fournier S, et al. Impact of Transplant-Related Stressors and Feelings of Indebtedness on Psychosocial Adjustment Following Kidney Transplantation. *Journal of Clinical Psychology in Medical Settings* 2004;11(1):63-73. doi: 10.1023/B:JOCS.0000016271.10786.ee
25. Chilcot J, Spencer BWJ, Maple H, et al. Depression and Kidney Transplantation. *Transplantation* 2014;97(7):717-21. doi: https://doi.org/10.1097/01.TP.0000438212.72960.ae
26. Shemesh Y, Peles-Bortz A, Peled Y, et al. Feelings of indebtedness and guilt toward donor and immunosuppressive medication adherence among heart transplant (HTx) patients, as assessed in a cross-sectional study with the Basel Assessment of Adherence to Immunosuppressive Medications Scale (BAASIS). *Clinical Transplantation* 2017;31(10):e13053. doi: 10.1111/ctr.13053
27. Poole J, Ward J, Deluca E, et al. Grief and loss for patients before and after heart transplant. *Heart & lung : the journal of critical care* 2016;45(3):193. doi: 10.1016/j.hrtlng.2016.01.006
28. Suzuki A, Kenmochi T, Maruyama M, et al. Changes in quality of life in deceased versus living-donor kidney transplantations. *Transplant Proc* 2012;44(1):287-9. doi: https://doi.org/10.1016/j.transproceed.2011.11.056 [published Online First: 2012/02/09]
29. Fox RC. Through the lenses of biology and sociology: Organ replacement. In: Williams SJ, Birke L, Bendelow GA, eds. Debating Biology: Sociological reflection on health, medicine and society. London: Routledge 2003.
30. Pinter J, Hanson CS, Craig JC, et al. 'I feel stronger and younger all the time'—perspectives of elderly kidney transplant recipients: thematic synthesis of qualitative research. *Nephrol Dial Transplant* 2016;31(9):1531-40. doi: https://doi.org/10.1093/ndt/gfv463
31. Boaz A, Morgan M. Working to establish 'normality' post-transplant: a qualitative study of kidney transplant patients. *Chronic illness* 2014;10(4):247-58. doi: https://doi.org/10.1177/1742395313504789 [published Online First: 2013/09/28]
32. Cormier NR, Gallo-Cruz SR, Beard RL. Navigating the new, transplanted self: how recipients manage the cognitive risks of organ transplantation. *Sociology of health & illness* 2017;39(8):1496-513. doi: https://doi.org/10.1111/1467-9566.12610 [published Online First: 2017/10/11]
33. Been-Dahmen JMJ, Beck DK, Peeters MAC, et al. Evaluating the feasibility of a nurse-led self-management support intervention for kidney transplant recipients: a pilot study. *BMC nephrology* 2019;20(1):143. doi: https://doi.org/10.1186/s12882-019-1300-7 [published Online First: 2019/04/29]
34. Curcani M, Tan M. the factors affecting the qualilty of life of patients who have undergone kidney transplants *Pakistan Journal of Medicine* 2012;27(5):1092-97.
35. Muehrer RJ, Becker BN. Life after transplantation: new transitions in quality of life and psychological distress. *Semin Dial* 2005;18(2):124-31. doi: https://doi.org/10.1111/j.1525-139X.2005.18214.x [published Online First: 2005/03/18]
36. National Institute for Health and Clinical Excellence, CG138 Patient experience in NHS services: improving the experience of care for people using adult NHS services Manchester: National Institute for Health and Clinical Excellence 2012 [Available from: http://guidance.nice.org.uk/cg138 accessed July 2019.
37. Damery S, Sein K, Combes G. The prevalence, experience and management of mild to moderate distress in patients with end stage renal disease: results from a multi-centre, mixed methods research study: NIHR CLAHRC-WM, University of Birmingham, 2019.
38. Been-Dahmen JMJ, Grijpma JW, Ista E, et al. Self-management challenges and support needs among kidney transplant recipients: A qualitative study. *Journal of advanced nursing* 2018;74(10):2393-405. doi: https://doi.org/10.1111/jan.13730 [published Online First: 2018/06/06]
39. Grijpma JW, Tielen M, van Staa AL, et al. Kidney transplant patients' attitudes towards self-management support: A Q-methodological study. *Patient Educ Couns* 2016;99(5):836-43. doi: https://doi.org/10.1016/j.pec.2015.11.018 [published Online First: 2015/12/20]

For peer review only

For peer review only - http://bmjopen.bmj.com/site/about/guidelines.xhtml

COREQ (COnsolidated criteria for REporting Qualitative research) Checklist

A checklist of items that should be included in reports of qualitative research. You must report the page number in your manuscript where you consider each of the items listed in this checklist. If you have not included this information, either revise your manuscript accordingly before submitting or note N/A.

Topic	Item No.	Guide Questions/Description	Reported on Page No.
**Domain 1: Research team and reflexivity**			
Personal characteristics			
Interviewer/facilitator	1	Which author/s conducted the interview or focus group?	6
Credentials	2	What were the researcher's credentials? E.g. PhD, MD	6
Occupation	3	What was their occupation at the time of the study?	6
Gender	4	Was the researcher male or female?	6
Experience and training	5	What experience or training did the researcher have?	6
Relationship with participants			
Relationship established	6	Was a relationship established prior to study commencement?	6
Participant knowledge of the interviewer	7	What did the participants know about the researcher? e.g. personal goals, reasons for doing the research	6
Interviewer characteristics	8	What characteristics were reported about the inter viewer/facilitator? e.g. Bias, assumptions, reasons and interests in the research topic	6
**Domain 2: Study design**			
Theoretical framework			
Methodological orientation and Theory	9	What methodological orientation was stated to underpin the study? e.g. grounded theory, discourse analysis, ethnography, phenomenology, content analysis	Referred to prc
Participant selection			
Sampling	10	How were participants selected? e.g. purposive, convenience, consecutive, snowball	6
Method of approach	11	How were participants approached? e.g. face-to-face, telephone, mail, email	6
Sample size	12	How many participants were in the study?	8
Non-participation	13	How many people refused to participate or dropped out? Reasons?	Data not avala
Setting			
Setting of data collection	14	Where was the data collected? e.g. home, clinic, workplace	7
Presence of non-participants	15	Was anyone else present besides the participants and researchers?	Data not availa
Description of sample	16	What are the important characteristics of the sample? e.g. demographic data, date	8
Data collection			
Interview guide	17	Were questions, prompts, guides provided by the authors? Was it pilot tested?	7
Repeat interviews	18	Were repeat inter views carried out? If yes, how many?	N/A
Audio/visual recording	19	Did the research use audio or visual recording to collect the data?	7
Field notes	20	Were field notes made during and/or after the inter view or focus group?	7
Duration	21	What was the duration of the inter views or focus group?	7
Data saturation	22	Was data saturation discussed?	19
Transcripts returned	23	Were transcripts returned to participants for comment and/or	7

For peer review only

For peer review only - http://bmjopen.bmj.com/site/about/guidelines.xhtml

Topic	Item No.	Guide Questions/Description	Reported on Page No.
		correction?	
**Domain 3: analysis and findings**			
Data analysis			
Number of data coders	24	How many data coders coded the data?	7
Description of the coding tree	25	Did authors provide a description of the coding tree?	9
Derivation of themes	26	Were themes identified in advance or derived from the data?	7
Software	27	What software, if applicable, was used to manage the data?	7
Participant checking	28	Did participants provide feedback on the findings?	7
Reporting			
Quotations presented	29	Were participant quotations presented to illustrate the themes/findings? Was each quotation identified? e.g. participant number	9-16
Data and findings consistent	30	Was there consistency between the data presented and the findings?	9-16
Clarity of major themes	31	Were major themes clearly presented in the findings?	9-16
Clarity of minor themes	32	Is there a description of diverse cases or discussion of minor themes?	10

Developed from: Tong A, Sainsbury P, Craig J. Consolidated criteria for reporting qualitative research (COREQ): a 32-item checklist for interviews and focus groups. *International Journal for Quality in Health Care*. 2007. Volume 19, Number 6: pp. 349 – 357

Once you have completed this checklist, please save a copy and upload it as part of your submission. DO NOT include this checklist as part of the main manuscript document. It must be uploaded as a separate file.

For peer review only
